# Baseline plasma KL-6 level predicts adverse outcomes in patients with idiopathic pulmonary fibrosis receiving nintedanib: a retrospective real-world cohort study

**DOI:** 10.1186/s12890-021-01530-6

**Published:** 2021-05-15

**Authors:** Tang-Hsiu Huang, Chin-Wei Kuo, Chian-Wei Chen, Yau-Lin Tseng, Chao-Liang Wu, Sheng-Hsiang Lin

**Affiliations:** 1 Division of Chest Medicine, Department of Internal Medicine, National Cheng Kung University Hospital, College of Medicine, National Cheng Kung University, Tainan, Taiwan; 2 Institute of Clinical Medicine, College of Medicine, National Cheng Kung University, Tainan, Taiwan; 3 Division of Thoracic Surgery, Department of Surgery, National Cheng Kung University Hospital, College of Medicine, National Cheng Kung University, Tainan, Taiwan; 4 Department of Biochemistry and Molecular Biology, College of Medicine, National Cheng Kung University, Tainan, Taiwan; 5 Department of Public Health, College of Medicine, National Cheng Kung University, Tainan, Taiwan; 6 Biostatistics Consulting Center, National Cheng Kung University Hospital, College of Medicine, National Cheng Kung University, Tainan, Taiwan

**Keywords:** Nintedanib, Krebs von den Lungen-6, Surfactant protein A, Diffusion capacity for carbon monoxide, Acute exacerbation, Mortality

## Abstract

**Background:**

Nintedanib is effective for treating idiopathic pulmonary fibrosis (IPF), but some patients may exhibit a suboptimal response and develop on-treatment acute exacerbation (AE-IPF), hepatic injury, or mortality. It remains unclear which patients are at risk for these adverse outcomes.

**Methods:**

We analysed the demographic and clinical data, baseline plasma levels of Krebs von den Lungen-6 (KL-6) and surfactant protein A (SPA), and longitudinal clinical courses of a real-world cohort of IPF patients who received nintedanib ≥ 14 days between March 2017 and December 2020. Cox proportional-hazards regression, subdistribution hazards regression, and sensitivity analyses were performed to investigate the association between baseline predictors and AE-IPF, mortality, and nintedanib-related hepatic injury. The relationship between baseline predictors and pulmonary function decline was determined.

**Results:**

Fifty-seven patients were included, of whom 24 (42%) developed hepatic injury, 20 (35%) had AE-IPF, and 16 (28%) died on-treatment. A baseline plasma KL-6 level ≥ 2.5 ng/mL, and diffusion capacity for carbon monoxide (D_LCO_) < 55% predicted, were associated with increased risk of hepatic injury (adjusted hazard ratio [aHR] was 3.46; 95% CI 1.13–10.60; *p* = 0.029 for KL-6, and 6.05; 95% CI 1.89–19.32; *p* = 0.002 for D_LCO_). Both factors also predicted severe and recurrent hepatic injury. Patients with baseline KL-6 ≥ 2.5 ng/mL also had a higher risk of AE-IPF (aHR 4.52; 95% CI 1.63–12.55; *p* = 0.004). For on-treatment mortality, baseline KL-6 ≥ 3.5 ng/mL and SPA ≥ 600 pg/mL were significant predictors (aHR 5.39; 95% CI 1.16–24.97; *p* = 0.031 for KL-6, and aHR 12.28; 95% CI 2.06–73.05; *p* = 0.006 for SPA). Results from subdistribution hazard regression and sensitivity analyses supported these findings. Patients with elevated baseline plasma KL-6 levels also exhibited a trend towards faster pulmonary function decline.

**Conclusions:**

For patients with IPF who are receiving nintedanib, we have identified baseline predictors, in particular plasma KL-6 levels, for the risk of adverse outcomes. Patients with these predictors may require close monitoring for unfavourable responses during treatment. Our findings also support the prognostic role of molecular markers like KL-6 and may contribute to future formulation of more individualized therapeutic strategies for IPF.

**Supplementary Information:**

The online version contains supplementary material available at 10.1186/s12890-021-01530-6.

## Introduction

Idiopathic pulmonary fibrosis (IPF) is a rare but progressive and devastating interstitial lung disease [1–3]. The median survival after diagnosis is around three years, worse than that of many cancers [2–4]. Patients with IPF are also at risk of developing acute exacerbation, which is characterized by extensive alveolar injury and accelerated fibrosis, leading to rapid and severe respiratory deterioration and even death [2, 3, 5, 6]. The pathogenesis of IPF is not fully understood, but much progress has been achieved particularly over the last decade [3]. Two anti-fibrotic agents with proven efficacy, nintedanib and pirfenidone, have recently become available [7, 8]. Nintedanib is a competitive tyrosine kinase inhibitor, interrupting pathways downstream of profibrotic growth factors [3, 9]. Accumulating evidence reveals that nintedanib slows the decline in forced vital capacity and lowers the risk of acute exacerbation [4, 10]. Pooled data from the initial major trials even revealed a possible reduction in the risk of on-treatment mortality [10].

Despite this, in both trials and real-world experience from a range of countries and ethnic groups, the therapeutic efficacy of nintedanib has not been universal: some patients receiving nintedanib might still exhibit a rapid decline in the pulmonary functions or develop acute exacerbation and even died [11–32]. In addition, nintedanib causes adverse reactions, the most common of which are gastrointestinal complaints; hepatotoxicity is also a concern. Hepatic injury during nintedanib therapy has been consistently described in clinical trials [7, 10–14] and post-marketing reports [15–32]. Relatively high incidence rates of hepatitis have been reported particularly from East Asia [19, 20, 26–28]. Nintedanib-related hepatic injury potentially interrupts the therapy and compromises first-pass metabolism, increasing exposure to the drug and thus the risks of other adverse reactions [33–35].

Pulmonary function parameters and radiographic features on computed tomography images are currently important determinants for the diagnosis and severity and prognosis assessment for IPF. However, accumulating evidence shows that blood levels of certain lung-specific macro-molecules, such as Krebs von den Lungen-6 (KL-6, also known as mucin-1, a glycoprotein expressed on the cell membrane of type 2 alveolar cells) and surfactant protein A (SPA, also secreted by type 2 alveolar cells), are also correlated with the severity of fibrosis and may serve as biomarkers for determining the clinical aspects of IPF [36–40]. While pulmonary function parameters are commonly used to assess the efficacy of antifibrotic treatment, the utility of these, as well as clinical and bio-chemical, characteristics for predicting therapeutic response and adverse outcomes during antifibrotic treatment remains to be investigated. In this study, we hypothesized that baseline characteristics, including blood levels of KL-6 and SPA, of patients with IPF could predict the risk of three important adverse outcomes (on-treatment acute exacerbation, mortality, and hepatic injury) during nintedanib treatment.

## Methods

### Study design and population

This retrospective cohort study involved patients with IPF at National Cheng Kung University Hospital (NCKUH) who had been treated with nintedanib and received regular follow-ups from 1 March, 2017, until 31 December, 2020. The study was approved by the NCKUH institutional review board (B-ER-105-390 and A-ER-107-193). The inclusion criteria for this study were as follows: naïve to anti-fibrotic therapy, aged > 50 years, diagnosed with IPF according to the international guidelines [41, 42] and based on a multi-disciplinary approach, and received uninterrupted nintedanib therapy for ≥ 14 days. We followed the patients until death or 31 December, 2020. Drug compliance was assessed upon each return visit. The electronic medical records of every patient were carefully reviewed and the following data were collected: baseline demographics, comorbidities (which also included the echocardiographic evidence of pulmonary hypertension, and factors necessary for the calculation of the Charlson comorbidity index), serial pulmonary function parameters (recorded at least eight weeks apart), gender-age-physiology (GAP) stages, and pertinent clinical data (including all medications of which the patient took at least three doses concurrently with nintedanib treatment within two weeks before each blood test for hepatic enzymes). We also checked the baseline plasma levels of KL-6 and SPA shortly before nintedanib treatment began, via sandwich enzyme-linked immunosorbent assay (ELISA), using specialized kits from Fine Test (Wuhan Fine Biotech, Wuhan City, China). The protocols for specimen processing and the ELISA are available in Additional file 1: Appendix A.

### Important definitions

We focused on three major on-treatment adverse outcomes: acute exacerbation, mortality, and nintedanib-related hepatic injury. The index date was the date when the first dose of nintedanib was taken. “On-treatment” refers to the period between the index date and either day 28 after the last dose of nintedanib [7] (for those who prematurely discontinued the therapy) or 31 December, 2020 (for those who continued the therapy). Patients on the “full dose” took 150 mg of nintedanib twice daily, while patients on the “reduced dose” (for managing drug-related adverse effects) took 100 mg daily, 150 mg daily, or 100 mg twice daily, at the discretion of the treating pulmonologist. Acute exacerbation of IPF (AE-IPF) was defined according to the recent international working group report, which specifically excludes events with identifiable infectious or non-infectious causes of acute deterioration [6]. We defined “mortality” as all-cause mortality. Nintedanib-related hepatitis injury was strictly defined as a blood alanine transaminase (ALT) level above the upper limit of normal (ULN, < 50 IU/L at NCKUH), with or without associated hyperbilirubinemia or symptoms, that could not be attributed to any other aetiology, which occurred while the patient was still receiving nintedanib treatment [26, 27, 43]. Severe hepatic injury was defined as an increase in blood ALT level to ≥ 3 × ULN. Patients were defined as having recurrent hepatic injury if they had already experienced nintedanib-related hepatic injury and recovered after temporary discontinuation or dose reduction of nintedanib, but then again developed unexplained ALT elevation above the ULN upon resumption of nintedanib treatment. Echocardiographic evidence of pulmonary hypertension was defined as an estimated systolic pulmonary arterial pressure (which was derived from tricuspid regurgitation jet velocity) ≥ 35 mmHg [44]. Serial differences in pulmonary function parameters were standardized into 24-week and annual (52-week) rates of change using the following formulae:

24-week rate of change:

$$\begin{aligned} & \left[ {\left( {{\text{FVC}}_{{{\text{last}}}} {-}{\text{FVC}}_{{{\text{baseline}}}} } \right)/\left( {{\text{time}}\,{\text{interval}}\,{\text{in}}\,{\text{weeks}}} \right)} \right] \times 24 \\ & \left[ {\left( {{\text{D}}_{{{\text{LCO}}\,{\text{last}}}} {-}{\text{D}}_{{{\text{LCO}}\,{\text{baseline}}}} } \right)/\left( {{\text{time}}\,{\text{interval}}\,{\text{in}}\,{\text{weeks}}} \right)} \right] \times 24 \\ \end{aligned}$$

Annual (52-week) rate of change:

$$\begin{aligned} & \left[ {\left( {{\text{FVC}}_{{{\text{last}}}} {-}{\text{FVC}}_{{{\text{baseline}}}} } \right)/\left( {{\text{time}}\,{\text{interval}}\,{\text{in}}\,{\text{weeks}}} \right)} \right] \times 52 \\ & \left[ {\left( {{\text{D}}_{{{\text{LCO}}\,{\text{last}}}} {-}{\text{D}}_{{{\text{LCO}}\,{\text{baseline}}}} } \right)/\left( {{\text{time}}\,{\text{interval}}\,{\text{in}}\,{\text{weeks}}} \right)} \right] \times 52 \\ \end{aligned}$$

where FVC represents forced vital capacity and D_LCO_ represents diffusion capacity of the lung for carbon monoxide. The subscript “first” indicates measurements that were closest in time before the initiation of nintedanib treatment, and the subscript “last” indicates measurements that were closest in time before the last dose of nintedanib (for patients who prematurely discontinued the therapy) or 31 December, 2020 (for patients who continued the therapy).

### Statistical analysis

Categorical data are presented as counts and percentages, and continuous variables are presented as mean (standard deviation) or median (interquartile range [IQR]) if not normally distributed. No imputation of missing data was made. Variables were compared between patient groups using Fischer’s exact test or the Mann–Whitney U test, whichever was more appropriate. The optimal cut-off value of a continuous variable was determined using receiver operating characteristic (ROC) curve, based on the combined consideration of the area under the curve, accuracy, and Youden’s index. The assumption of proportional hazards was checked using the Shoenfeld test. Cox proportional-hazards regression models were constructed to assess the performance of candidate risk factors in longitudinally predicting the risks of adverse outcomes. When we conducted multi-variable regression analyses, in addition to including the candidate predictors, we routinely adjusted for the baseline GAP stage, Charlson comorbidity index, and duration of nintedanib treatment. For acute exacerbation and hepatic injury, the competing risk of on-treatment mortality was further controlled for by subdistribution hazard regression. Sensitivity analyses were performed to assess the robustness of all the multi-variable models constructed. All tests were two-tailed, and a *p*-value < 0.05 was considered statistically significant. Statistical analyses were performed using R (Version 3.6.1) and SPSS (Version 22, SPSS, USA). Graphs were plotted using MedCal (Version 16.8.4, MedCal Software, Belgium).

## Results

**Fig. 1:**
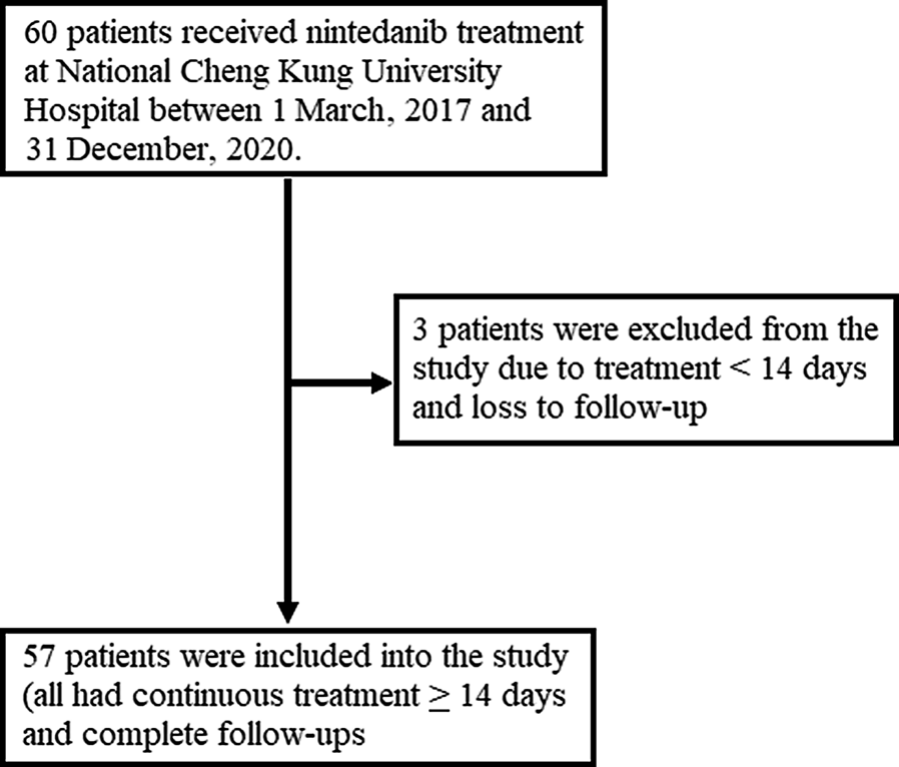
The inclusion and exclusion flowchart for this study

Sixty patients received nintedanib treatment and were followed-up at NCKUH from 1 March, 2017, until 31 December, 2020. Three patients were excluded due to incomplete follow-ups; the remaining 57 were included in this study. Figure 1 shows the inclusion and exclusion flowchart for this study.

Table 1 summarizes the baseline characteristics and adverse outcomes of all the included patients. The study cohort was predominantly male and had a mean age of 75.4 ± 9.4 years. Fifteen (26%) patients exhibited sonographic evidence of fatty liver before they began receiving nintedanib, but none had cirrhosis, and all had normal levels of hepatic enzymes at baseline. The median duration of nintedanib treatment was 345 days (IQR, 91–706 days). Overall, 24 (42%) patients developed hepatic injury, manifesting as an asymptomatic elevation in the blood ALT level without concurrent hyperbilirubinemia. Eight patients had severe injury (ALT ≥ 3 × ULN). All the hepatic injury events were nonfatal and spontaneously resolved following transient reduction of the nintedanib dosage or temporary withdrawal of nintedanib, although 14 patients developed recurrent hepatic injury after resumption of nintedanib treatment. Twenty (35%) patients had on-treatment AE-IPF, and 16 (28%) patients died on treatment. Regarding the timing of the first onset of these adverse outcomes, all hepatic injury occurred within one year of the commencement of treatment, and in all cases the onset of AE-IPF occurred within three years of the commencement of treatment. Of the patients who died on-treatment, six (38%) did so within the first year. All the on-treatment mortality events occurred within four years of the commencement of treatment (Additional file 1: Table S1). It is worth noting that in those patients who developed both hepatic injury and AE-IPF, all AE-IPF events occurred after the hepatic injury.

**Table 1:** Baseline characteristics and adverse outcomes of the 57 patients

Baseline characteristics and outcome events	Results
Age, years	75.4 ± 9.4
Sex	
Female, n (%)	9 (16)
Male, n (%)	48 (84)
Body height, cm	162.2 ± 7.7
Body weight, kg	63.5 ± 10.8
Body mass index, kg/m^2^	24.0 (21.5 to 26.5)
Body surface area, m^2^	1.67 ± 0.15
Charlson comorbidity index	5 (3 to 6)
Echocardiographic evidence of pulmonary hypertension, n (%)	36 (63)
Echocardiographic evidence of LV dysfunction, n (%)	2 (4)
Dyslipidaemia, n (%)	26 (46)
Chronic hepatitis B, n (%)	6 (11)
Chronic hepatitis C, n (%)	5 (9)
Other non-viral liver condition, n (%)	3 (5)
Fatty liver on baseline sonography, n (%)	15 (26)
Cigarette smoking status	
Never smoker, n (%)	26 (46)
Current smoker, n (%)	4 (7)
Former smoker, n (%)	27 (47)
Baseline plasma KL-6 level, ng/mL	1.48 (0.55 to 2.82)
Baseline plasma SPA level, pg/mL	283.6 (157.3 to 435.4)
Baseline oximetry breathing ambient air, %	95 (93 to 97)
Baseline D_LCO_, mmol/min/kPa	2.78 (2.11 to 3.96)
Baseline D_LCO_, % predicted	55 (38 to 70)
Baseline FVC, L	2.02 ± 0.49
Baseline FVC, % predicted	67 ± 12
Stages based on the GAP index	
Stage 1, n (%)	14 (25)
Stage 2, n (%)	31 (54)
Stage 3, n (%)	12 (21)
Nintedanib-related hepatic injury, n (%)	24 (42)
On-treatment AE-IPF, n (%)	20 (35)
On-treatment mortality, n (%)	16 (28)
Duration of nintedanib therapy, days	345 (91 to 706)
Time to first nintedanib-related hepatic injury, days	69 (17 to 156)
Time to first on-treatment AE-IPF, days	238 (111 to 431)
Time to on-treatment mortality, days	486 (217 to 811)
Time between plasma sampling and nintedanib initiation, days	6 (0 to 28)
Time between baseline pulmonary functions and nintedanib initiation, days	28 (16 to 51)
Time between plasma sampling and baseline pulmonary functions, days	24 (12 to 81)
Annual rate of change in FVC, L/52 weeks	− 0.13 (− 0.26 to + 0.06)
Annual rate of change in D_LCO_, % predicted/52 weeks	− 9 (− 29 to − 2)

Categorical data are presented as counts and percentages, and continuous variables were presented as mean (± standard deviation) or median (interquartile range) if non-normally distributed. AE-IPF, acute exacerbation of idiopathic pulmonary fibrosis; D_LCO_, diffusion capacity for carbon monoxide; FVC, forced vital capacity; GAP, gender, age, physiology; KL-6, Krebs von den Lungen-6; LV, left ventricular; SPA, surfactant protein A

Compared to patients without hepatic injury, patients who developed hepatic injury had significantly higher baseline levels of plasma KL-6 (2.72 ng/mL [IQR 1.82–4.05] and 0.94 ng/mL [IQR 0.44–1.63], respectively; *p* < 0.001) and significantly lower D_LCO_ (42% predicted [IQR 31–54] and 60% predicted [IQR 53–83], respectively; *p* = 0.001). Patients with hepatic injury also exhibited a borderline-significant pattern of higher plasma SPA, lower pulse oximetry (breathing ambient air), and higher frequency of pulmonary hypertension (Additional file 1: Table S2). No significant difference was identified in the dosing, the time to the first test for hepatic enzymes, or the concurrently used medication (Additional file 1: Table S3) between patients with and without hepatic injury. Patients who had on-treatment AE-IPF also had significantly higher plasma levels of KL-6 (3.11 ng/mL [IQR 1.38–5.07] versus 1.03 ng/mL [IQR 0.48–1.86]; *p* = 0.001) and SPA (412.6 pg/mL [IQR 181.8–478.5] versus 235.6 pg/mL [IQR 157.3–379.9]; *p* = 0.042) than those who did not have AE-IPF. They also had a borderline-significant pattern of a higher frequency of pulmonary hypertension than patients without AE-IPF (Additional file 1: Table S4). The 16 patients who died on-treatment had significantly higher baseline plasma levels of KL-6 (3.61 ng/mL [IQR 1.28–8.22] versus 1.31 ng/mL [IQR 0.48–2.20]; *p* = 0.001) and SPA (447.9 pg/mL [IQR 393.5–697.3] versus 226.0 pg/mL [IQR 140.2–373.7]; *p* < 0.001) than those who survived. They also had significantly higher frequencies of pulmonary hypertension (88% versus 54%; *p* = 0.03) and on-treatment AE-IPF (75% versus 20%; *p* < 0.001) than patients who survived (Additional file 1: Table S5). These variables were thus selected as candidate predictors for further analyses. The results from the ROC analysis for selecting cut-off values for continuous-variable candidate predictors are summarized in Additional file 1: Table S6. It is worth noting that there was no significant difference between patients with and without adverse outcomes in either their dosing regimen or the time intervals between blood sampling, baseline pulmonary functions, or the initiation of nintedanib treatment (Additional file 1: Tables S2, S4, S5).

**Fig. 2:**
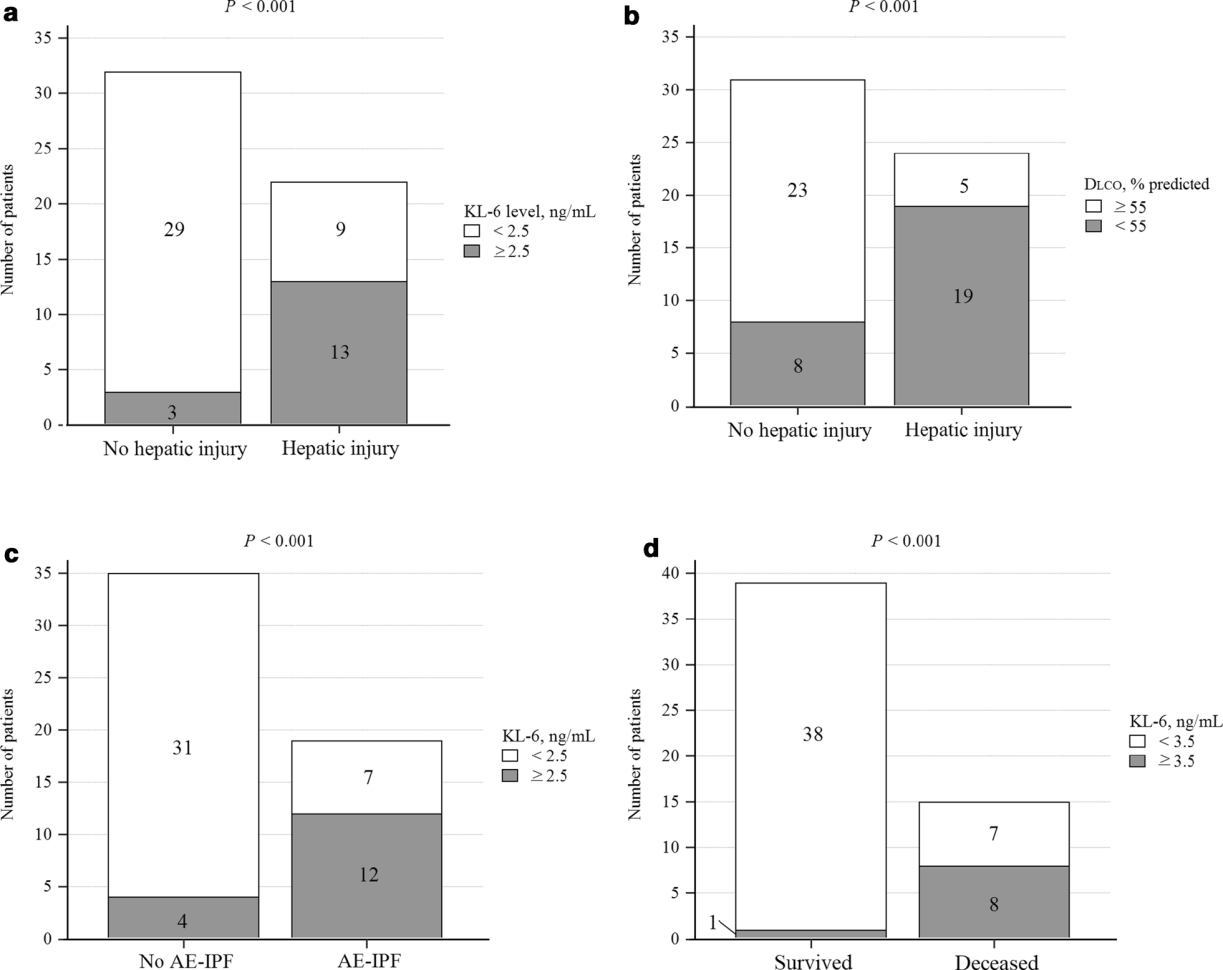
Distribution of patients above and below the cut-off values of the major predictors for the three adverse outcomes. Numbers within the bars are the patient counts. **a** KL-6 level versus nintedanib-related hepatic injury, **b** D_LCO_ % predicted versus nintedanib-related hepatic injury, **c** KL-6 level versus on-treatment AE-IPF, **d** KL-6 level versus on-treatment mortality, and **e** SPA level versus on-treatment mortality. *p*-values were derived from Fisher’s exact test. Number of patients with available data: 54 for KL-6, 55 for D_LCO_ % predicted, and 55 for SPA. *Abbreviations* AE-IPF, acute exacerbation of idiopathic pulmonary fibrosis; D_LCO_, diffusion capacity for carbon monoxide (in % predicted); KL-6, Krebs von den Lungen-6; SPA, surfactant protein A

**Fig. 3:**
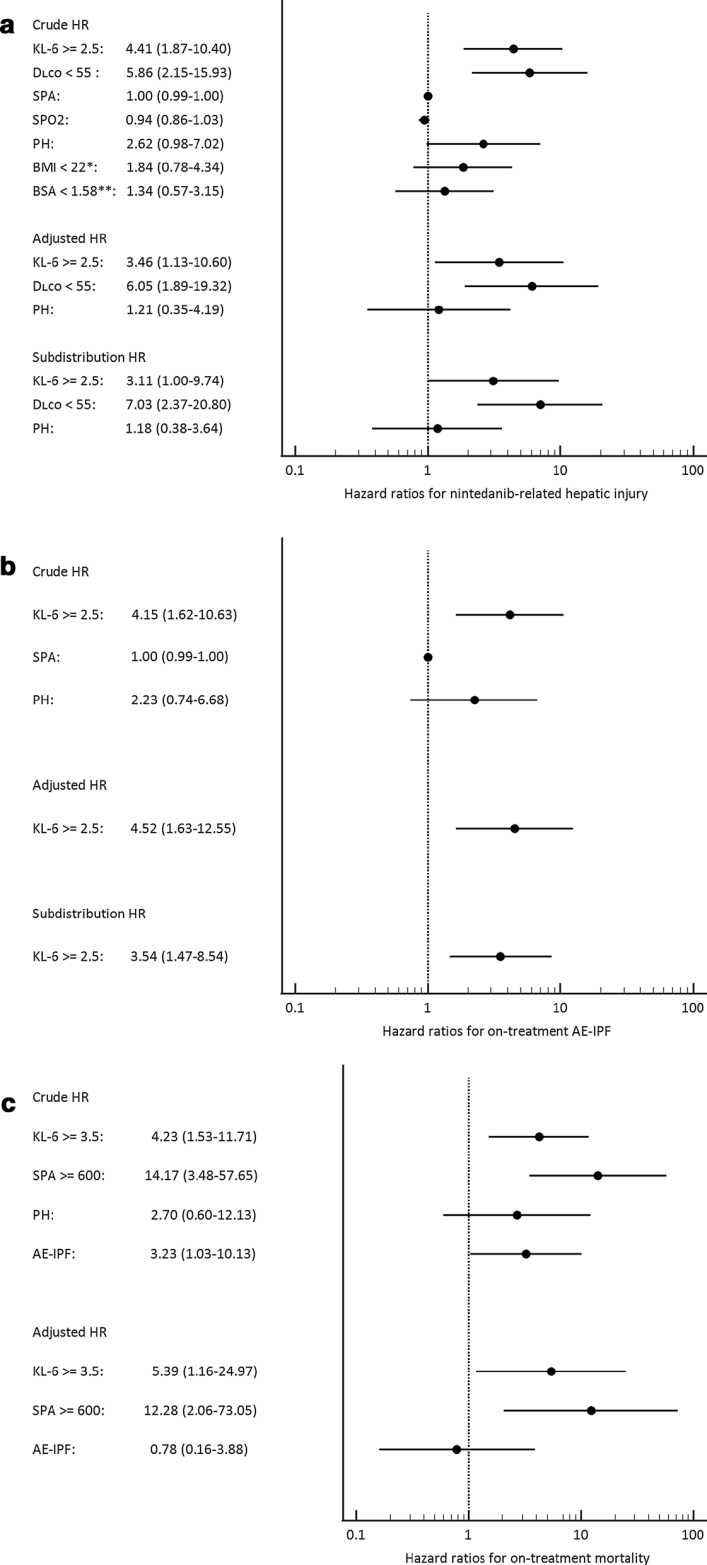
Forest plots showing the results of Cox proportional-hazards regression and subdistribution hazard regression analyses of candidate predictors for **a** nintedanib-related hepatic injury, **b** on-treatment acute exacerbation of IPF, and **c** on-treatment mortality. In addition to the candidate predictors shown, all the multi-variable regression models were also adjusted for gender-age-physiology (GAP) stage, Charlson comorbidity index, and treatment duration. *Abbreviation and Notes* AE-IPF, acute exacerbation of idiopathic pulmonary fibrosis; BMI, body mass index; BSA, body surface area; D_LCO_, diffusion capacity for carbon monoxide (in % predicted); HR, hazard ratio; KL-6, Krebs von den Lungen-6; PH, echocardiographic pulmonary hypertension; SPA, surfactant protein A; SPO2, pulse oximetry (while breathing ambient air). *This cut-off value was proposed by Ikeda et al. [26]. **This cut-off value was proposed by Ikeda et al. [27]

In the univariate and multi-variable Cox proportional-hazards regression analyses, only two candidate predictors (KL-6 ≥ 2.5 ng/mL and D_LCO_ < 55% predicted; Fig. 2a, b show the distribution of patients with respect to these cut-off values) consistently yielded significantly elevated crude and adjusted hazard ratios for nintedanib-related hepatic injury (the adjusted hazard ratio [aHR] for KL-6 was 3.46 [95% CI 1.13–10.60], *p* = 0.029; and the aHR for D_LCO_ was 6.05 [95% CI 1.89–19.32], *p* = 0.002). Pulmonary hypertension yielded a borderline-significant crude hazard ratio, but that failed to reach statistical significance in the multi-variable model (Fig. 3a and Additional file 1: Table S7). A baseline KL-6 ≥ 2.5 ng/mL and D_LCO_ < 55% predicted was also correlated with an increased risk of severe and recurrent hepatic injury during nintedanib treatment. For severe hepatic injury, the adjusted odds ratio (aOR) from the multi-variable ordinal logistic regression analysis was 9.58 (95% CI 1.97–55.67; *p* = 0.007) for KL-6 and 10.89 (95% CI 2.63–54.25; *p* = 0.002) for D_LCO_. For recurrent hepatic injury, the aOR was 26.01 (95% CI 4.19–260.13; *p* = 0.001) for KL-6 and 60.28 (95% CI 8.30–823.62; *p* < 0.001) for D_LCO_ (Additional file 1: Table S8). Ikeda et al. reported that patients with BMI < 22 kg/m^2^ or BSA < 1.58 m^2^ would have an increased risk for nintedanib-related hepatitis [26, 27]. However, we found no significant differences in BMI or BSA between patients with and without hepatic injury (Additional file 1: Table S2). Univariate regression analyses involving BMI and BSA (using the cut-off values proposed by Ikeda et al.) also found no significant differences (Fig. 3a and Additional file 1: Tables S7 and S8). With the same cut-off value as for hepatic injury (2.5 ng/mL), the baseline plasma KL-6 level was the only significant predictor of on-treatment AE-IPF in both the univariate and the multi-variable Cox proportional-hazards regression (aHR 4.52; 95% CI 1.63–12.55; *p* = 0.004; Fig. 3b and Additional file 1: Table S9). Figure 2c shows the distribution of patients above and below this cut-off value. For on-treatment mortality, the baseline plasma KL-6 level was also a significant predictor, but with a cut-off value of 3.5 ng/mL (aHR 5.39; 95% CI 1.16–24.97; *p* = 0.031). Another significant predictor in the multi-variable model for on-treatment mortality was baseline plasma SPA level, with a cut-off value of 600 pg/mL (aHR 12.28; 95% CI 2.06–73.05; *p* = 0.006; Fig. 2d, e show the distribution of patients above and below these cut-off values, while Fig. 3c shows the results of the Cox proportional-hazards regression analyses). Although the patients who died on-treatment had a significantly higher frequency of pulmonary hypertension at baseline and AE-IPF on treatment, these two predictors were not statistically significant in the regression models (Fig. 3c and Additional file 1: Table S10). To control for the competing risk of on-treatment mortality, we performed a multi-variable subdistribution hazard regression analysis for hepatic injury and for on-treatment AE-IPF, which yielded concordant and supportive results (the lower parts of Fig. 3a, b, and the far-right panels of Additional file 1: Tables S7 and S9). In addition, sensitivity analyses showed that, even in the presence of an unidentified confounder, the above-mentioned multi-variable Cox models and the predictors thereby derived yielded significantly elevated hazard ratios for the corresponding adverse outcomes (Additional file 1: Figures S1a to S1e).

**Fig. 4:**
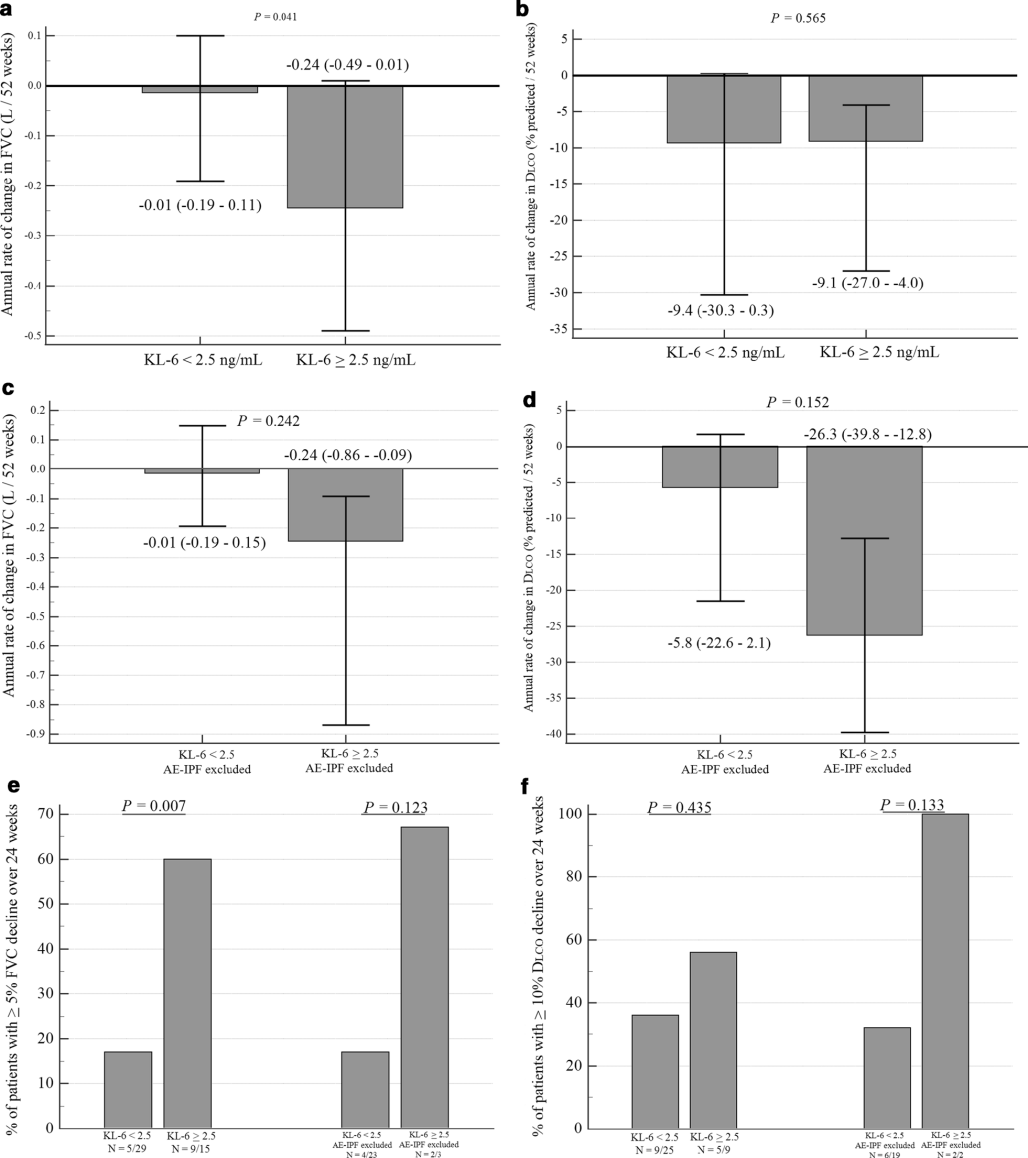
Comparison between patients with baseline plasma KL-6 < 2.5 ng/mL with patients with KL-6 ≥ 2.5 ng/mL in: **a** annual rate of FVC decline; **b** annual rate of D_LCO_ decline; **c** annual rate of FVC decline after excluding patients with AE-IPF; **d** annual rate of D_LCO_ decline after excluding patients with AE-IPF; **e** proportion of patients with ≥ 5% FVC decline over 24 weeks; **f** proportion of patients with ≥ 10% D_LCO_ decline over 24 weeks. In panels **a** to **d**, data are presented as medians with inter-quartile ranges, and *p*-values were derived from the Mann Whitney U test. In panels **e** and **f**, data shown are proportions of patients in each subgroup, and *p*-values were derived from Fisher’s exact test. *Abbreviation* AE-IPF, acute exacerbation of idiopathic pulmonary fibrosis; D_LCO_, diffusion capacity for carbon monoxide; FVC, forced vital capacity; KL-6, Krebs von den Lungen-6

Because plasma KL-6 level was the only baseline factor that significantly predicted the risk of all three adverse outcomes, we further explored the relationship between KL-6 level and treatment response in terms of pulmonary function decline. Forty-four and 34 patients had at least one follow-up FVC and D_LCO_ assessment, respectively, at least eight weeks later and while still on-treatment. For FVC, 40 patients had a time interval between the baseline and the last measurements of more than six months, of whom the time interval was more than one year in 25 patients (median interval, 409 days; IQR, 265–712). For D_LCO_, 32 patients had a time interval between the measurements of more than six months, of whom the time interval was more than one year in 19 patients (median interval, 393 days; IQR, 280–767). The overall annual rate of change in FVC and D_LCO_ in the cohort was − 0.13 L (IQR, − 0.26 to + 0.06) and − 9% (IQR, − 29 to − 2), respectively (Table 1). However, patients with KL-6 ≥ 2.5 ng/mL exhibited a significantly higher annual rate of decline in FVC than patients with KL-6 < 2.5 ng/mL, but a similar annual rate of decline in D_LCO_ (Fig. 4a, b). A significantly higher proportion of patients with KL-6 ≥ 2.5 ng/mL had a decline in FVC of ≥ 5% over 24 weeks than patients with KL-6 < 2.5 ng/mL. For D_LCO_, a higher proportion of patient with KL-6 ≥ 2.5 ng/mL had a decline ≥ 10% over 24 weeks than patients with KL-6 < 2.5 ng/mL, although this difference was not statistically significant (left half of Fig. 4e, f). Similar patterns of pulmonary function decline were observed with a KL-6 cut-off value of 3.5 ng/mL (Additional file 1: Figures S2a to S2d). Even if we excluded patients with on-treatment AE-IPF from this analysis, we observed similar trends in pulmonary function decline, in addition to a nonsignificant pattern of a faster annual decrease in D_LCO_, in patients with KL-6 ≥ 2.5 ng/mL (Fig. 4c, d, and the right half of Fig. 4e, f).

## Discussions

In this study, we confirmed our hypothesis and found that for patients with IPF who receive nintedanib treatment, certain baseline markers predict the risk of on-treatment adverse outcomes. Specifically, patients with baseline plasma KL-6 levels ≥ 2.5 ng/mL had a higher risk of nintedanib-related hepatic injury (including severe and recurrent injury) and on-treatment acute-exacerbation of IPF. Patients with baseline plasma KL-6 levels ≥ 3.5 ng/mL had a higher risk of on-treatment mortality. Having a baseline D_LCO_ < 55% predicted and plasma SPA levels ≥ 600 pg/mL was also associated with increased risk of hepatic injury and on-treatment mortality, respectively. In addition, regardless of which cut-off values for KL-6 was used (2.5 or 3.5 ng/mL), a pattern of greater on-treatment decline in FVC and D_LCO_ was observed in patients whose baseline plasma KL-6 levels were above the cut-off value.

The recent introduction of antifibrotic therapy has been a breakthrough in the management of IPF. Although it is not a curative therapy and its efficacy for reducing mortality remains inconclusive, nintedanib has been shown to halt pulmonary function decline and is likely to reduce the risk of acute exacerbation. [13, 16, 19, 20]. However, not all patients receiving nintedanib exhibit the same favourable response. It remains unclear whether and how we can predict who will benefit most from this treatment and who will have a poor response or even develop adverse outcomes. On the other hand, nintedanib has been known to cause drug-induced liver injury ever since its early major trials [7, 10, 24, 45]. Hepatic impairment has also consistently been reported in real-world data from various countries (1.6–9.6% of treated patients) [15, 17, 18, 21–25], and as well as in the recent INPULSIS-ON and INBUILD trials [13, 14]. Late-onset hepatotoxicity has also been described [46]. Data from East Asia have shown relatively high incidence rates of hepatitis, reaching 67.6% [19, 20, 26–29]. In our study cohort, 42% of patients developed hepatic injury.

Functional parameters like FVC and D_LCO_ are important for diagnosing and assessing IPF [16, 20, 47, 48]. Serial decline in pulmonary function parameters is also associated with increasing risk of mortality [49, 50]. However, these parameters may be insensitive to early disease or minor progression, and they are also subject to variation resulting from suboptimal testing procedures and patient-specific factors [51–54]. The blood levels of circulating lung-specific macromolecules such as KL-6 [38, 39, 55–58] and SPA [37, 59] appear to correlate with the severity and prognosis of IPF. Yokoyama et al. retrospectively studied 23 patients with IPF and found that a high baseline serum KL-6 level (≥ 1000 U/mL) was associated with significantly diminished survival [56]. Using the same cut-off value, Wakamatsu et al. also showed that an initially high serum KL-6 level with a subsequent increasing pattern was associated with poor survival and a steep decline in FVC [57]. In addition, Ohshimo et al. proposed that a high baseline serum KL-6 level (≥ 1300 U/mL) predicts a subsequent risk of acute exacerbation [58]. However, none of the patients included in these studies received nintedanib or any other anti-fibrotic treatment. Bergantini et al. followed 23 patients with IPF who received nintedanib for 12 months and found that uninterrupted nintedanib treatment may have stabilized their serum KL-6 levels in serial tests, and that variation in serum KL-6 levels was correlated with serial variations in D_LCO_ [60]. Yoshikawa et al. analysed data from 49 patients receiving anti-fibrotic treatment (including 26 who were receiving nintedanib) and found that the patterns of change in serum SPA, surfactant protein D, and KL-6 levels three and six months into treatment were correlated with the rate of deterioration in FVC and D_LCO_ [61]. Building on these pioneering findings, the present study is novel in showing that the baseline levels of KL-6 in particular, and SPA, have a predictive role in patients with IPF who receive nintedanib treatment, not only for mortality and pulmonary function deterioration, but also for other clinically important outcomes like acute exacerbation and hepatic injury. The findings of our study highlight the heterogeneity of patients with IPF, identify subgroups of patients who may have a poor response and adverse outcomes despite ongoing nintedanib anti-fibrotic treatment, and further support the clinical utility of blood molecular markers (particularly KL-6) for IPF.

We suspect that the mechanism underlying the association between the predictors we have identified and the risk of the three adverse outcomes is related to the severity of lung parenchymal fibrotic destruction and overall physical frailty. The blood levels of KL-6 and SPA probably reveal the degree of injury and dysfunction of type 2 alveolar cells, and the associated fibrotic disruption of the alveolar-endothelial interface [39, 55]. Supporting this rationale is the fact that, when we stratified the cohort according to the level of plasma KL-6 using a cut-off value 2.5 ng/mL (as shown in Additional file 1: Table S11), we found that patients with KL-6 ≥ 2.5 ng/mL indeed had more severe physiological impairment and physical frailty than those with KL-6 < 2.5 ng/mL. This was reflected by differences in relevant clinical indices: significantly lower body weights, body surface area, and D_LCO_; significantly higher frequencies of pulmonary hypertension; and borderline-significant patterns of lower body mass index and higher frequencies of GAP-stage-3 disease. Interestingly, in the statistical analyses to identify predictors for on-treatment adverse outcomes, these clinical indices individually did not perform as well as KL-6 or, to a lesser extent, SPA. This can probably be explained by the fact that KL-6 and SPA indicate disease severity at the histological-molecular level. They are therefore less susceptible to confounding effects from comorbidities, and variations in function test procedures, than other clinical or functional indices. Considering that pharmacodynamically nintedanib can only slow down and not reverse the fibrogenesis [3, 9], it is plausible that patients with higher levels of KL-6 and SPA (indicating more severe fibrotic destruction) would be less likely to exhibit a strong therapeutic response and more likely to progress towards unfavourable outcomes. This proposed mechanism may also account for the enhanced risk of nintedanib-related hepatic injury. Patients with elevated KL-6 and/or low D_LCO_ probably already had advanced IPF and may have experienced frequent episodes of hypoxemia (either resting or exertion-induced). Hypoxemia can interfere with the oxygen-demanding steps of nintedanib metabolism (such as glucuronidation by UGT-enzymes) in hepatocytes, thereby retarding the clearance of potentially harmful metabolites [35, 62]. The higher risk of hepatic injury appears not to be the direct consequence of increased circulating KL-6 per se, because circulating mucins are cleared mainly by hepatic endothelial cells and Kupffer cells, rather than by hepatocytes [63]. Further research to ascertain whether our proposed mechanisms are valid would be helpful and inspiring.

This study has some limitations. Due to the overall rarity of IPF [2, 3, 64], the size of our study cohort is relatively small. Nevertheless, our findings, which are derived from a detailed real-world database with extended longitudinal follow-up, do provide insights into important clinical issues. In addition, we included only patients of Han Chinese ethnicity who were followed at a single tertiary center in Taiwan. Genetic polymorphisms of KL-6 (such as the rs4072037 single nucleotide polymorphism, which was not checked in the present study) have been described. These may result in different cut-off thresholds in different ethnicities for discriminating between patients with and without interstitial lung diseases (including IPF) [65]. It remains to be determined whether such polymorphisms may also effect the cut-off thresholds for predicting adverse outcomes. Furthermore, we did not include subsequent KL-6 measurements, and therefore could not determine the relationship between longitudinal trends in KL-6 levels and adverse clinical outcomes. Future research involving serial measurements of KL-6 and a larger population with greater diversity, e.g., in both ethnicity and pulmonary spirometry results, or including patients with other progressive fibrosing interstitial lung disease (PF-ILDs) [14, 66], would help to validate the generalizability of our findings and provide more information about the clinical utility of KL-6 levels.

## Conclusions

For patients with IPF receiving nintedanib treatment, their baseline plasma level of KL-6 predicted their risk of on-treatment acute exacerbation, mortality, and hepatic injury (including severe and recurrent injury). Patients with elevated baseline plasma KL-6 levels also exhibited a pattern of more rapid pulmonary function decline. Additionally, an initially high plasma SPA level and low D_LCO_ were also associated with adverse on-treatment outcomes. The findings of this study may help to identify patients for whom close monitoring for unfavourable responses during nintedanib treatment would be important. It may also contribute to the future formulation of more individualized therapeutic strategies and support the prognostic roles of blood molecular markers for IPF in real-world clinical practice.

## Supplementary Information

**Additional file 1.  Supplementary figures and tables and the protocols for processing  blood specimens and for performing the enzyme-linked immunosorbent assay (ELISA). **

## Data Availability

The de-linked datasets used and analysed during the current study are available from the corresponding author on reasonable request.

## Abbreviations

AE-IPF: Acute exacerbation of idiopathic pulmonary fibrosis
aHR: Adjusted hazard ratio
ALT: Alanine transaminase
aOR: Adjusted odds ratio
BMI: Body mass index
BSA: Body surface area
D_LCO_: Diffusion capacity for carbon monoxide
ELISA: Enzyme-linked immunosorbent assay
FVC: Forced vital capacity
IPF: Idiopathic pulmonary fibrosis
IQR: Inter-quartile range
KL-6: Krebs von den Lungen-6
LV: Left ventricular
ROC: Receiver operating characteristic
SPO2: Pulse oximetry (while breathing ambient air)
SPA: Surfactant protein A
ULN: Upper-limit of normal
95% CI: 95% confidence interval
