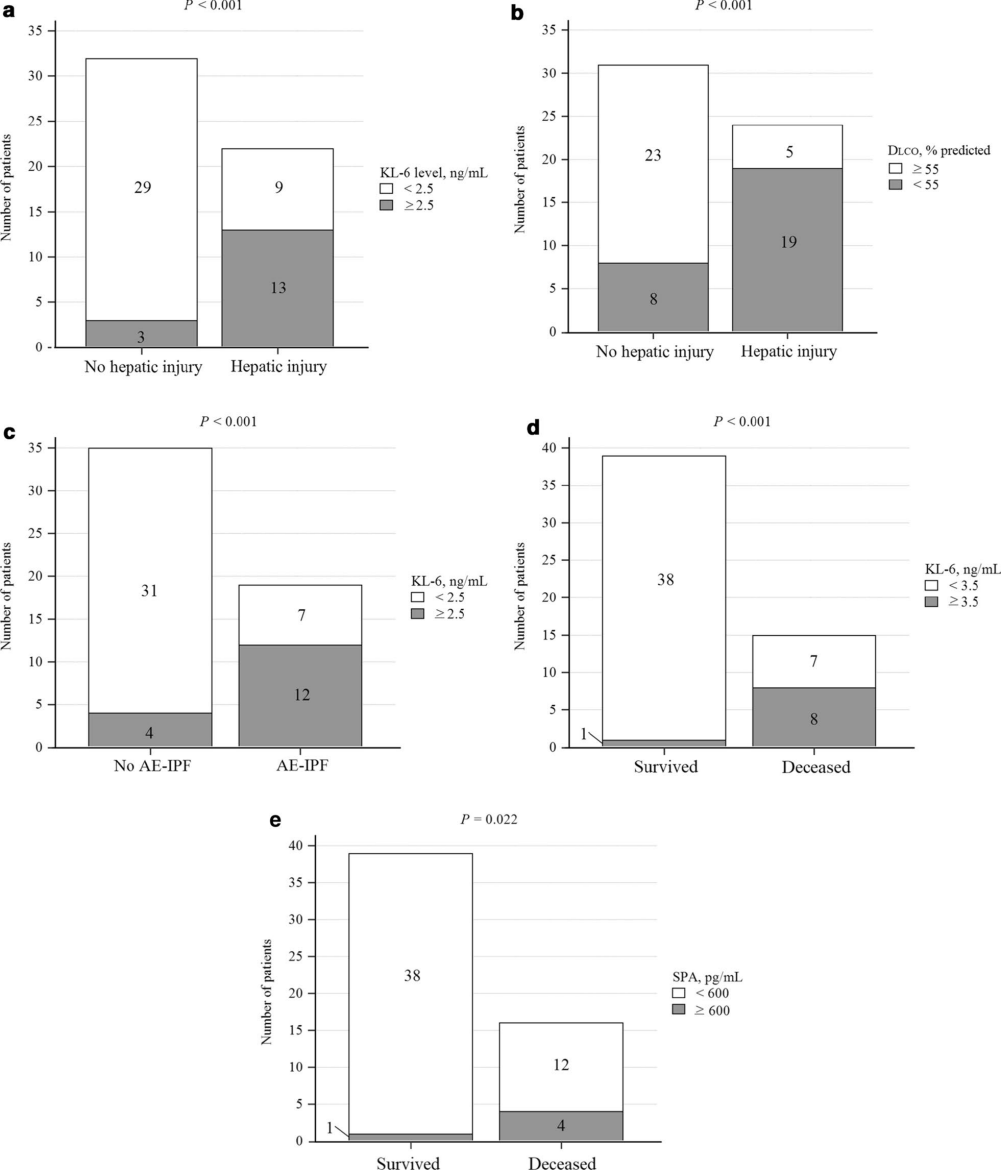# Correction to: Baseline plasma KL-6 level predicts adverse outcomes in patients with idiopathic pulmonary fibrosis receiving nintedanib: a retrospective real-world cohort study

**DOI:** 10.1186/s12890-021-01559-7

**Published:** 2021-06-08

**Authors:** Tang-Hsiu Huang, Chin-Wei Kuo, Chian-Wei Chen, Yau-Lin Tseng, Chao-Liang Wu, Sheng-Hsiang Lin

**Affiliations:** 1 Division of Chest Medicine, Department of Internal Medicine, National Cheng Kung University Hospital, College of Medicine, National Cheng Kung University, Tainan, Taiwan; 2 Institute of Clinical Medicine, College of Medicine, National Cheng Kung University, Tainan, Taiwan; 3 Division of Thoracic Surgery, Department of Surgery, National Cheng Kung University Hospital, College of Medicine, National Cheng Kung University, Tainan, Taiwan; 4 Department of Biochemistry and Molecular Biology, College of Medicine, National Cheng Kung University, Tainan, Taiwan; 5 Department of Public Health, College of Medicine, National Cheng-Kung University, Tainan, Taiwan; 6 Biostatistics Consulting Center, National Cheng Kung University Hospital, College of Medicine, National Cheng-Kung University, Tainan, Taiwan

## Correction to: BMC Pulm Med (2021) 21:165 https://doi.org/10.1186/s12890-021-01530-6

Following publication of the original article [1], it was brought to our attention that panel ‘e’ was missing from Fig. 2; the figure has since been corrected in the published article.

**Figure:**