# Supplementary material for: Baseline plasma KL-6 level predicts adverse outcomes in patients with idiopathic pulmonary fibrosis receiving nintedanib: a retrospective real-world cohort study
Source: BMC Pulm Med. 2021 May 15;21:165. doi: 10.1186/s12890-021-01530-6 (PMC8126113; doi:10.1186/s12890-021-01530-6)
Supplement: Supplementary file 1 — Additional file 1. Supplementary figures and tables and the protocols for processing blood specimens and for performing the enzyme-linked immunosorbent assay (ELISA). [file 12890_2021_1530_MOESM1_ESM.docx]

**Supplementary Material**

**Baseline plasma KL-6 level predicts adverse outcomes in patients with idiopathic pulmonary fibrosis receiving nintedanib – a retrospective real-world cohort study**

Tang-Hsiu Huang, MD^1,2^, Chin-Wei Kuo, MD^1,2^, Chian-Wei Chen, MD^1^, Yau-Lin Tseng, MD, PhD^3^, Chao-Liang Wu, PhD^4^, Sheng-Hsiang Lin, PhD^2,5,6^🖂

^1^ Division of Chest Medicine, Department of Internal Medicine, National Cheng Kung

University Hospital, College of Medicine, National Cheng Kung University, Tainan,

Taiwan

^2^ Institute of Clinical Medicine, College of Medicine, National Cheng Kung

University, Tainan, Taiwan

^3^ Division of Thoracic Surgery, Department of Surgery, National Cheng Kung

University Hospital, College of Medicine, National Cheng Kung University, Tainan,

Taiwan

^4^ Department of Biochemistry and Molecular Biology, College of Medicine, National

Cheng Kung University, Tainan, Taiwan

^5^ Department of Public Health, College of Medicine, National Cheng-Kung

University, Tainan, Taiwan

^6^ Biostatistics Consulting Center, National Cheng Kung University Hospital, College

of Medicine, National Cheng-Kung University, Tainan, Taiwan

**Table of Content for Supplementary Material**

| Page 3-5 | **Supplementary Figure S1** - Sensitivity analyses on the multi-variable Cox proportional-hazards regression models |
| --- | --- |
| Page 6 | **Supplementary Figure S2 -** Comparison of pulmonary functional decline rates between patient groups stratified by the plasma Krebs von den Lungen-6 levels ≥ or < 3.5 ng/mL |
| Page 7 | **Supplementary Table S1** - Timing of onset of on-treatment adverse outcomes |
| Page 8-9 | **Supplementary Table S2** - Baseline characteristics and outcome events of patients with and without nintedanib-related hepatic injury |
| Page 10-12 | **Supplementary Table S3 -** Medications used concurrently to nintedanib treatment by patients with and without hepatic injury |
| Page 13-14 | **Supplementary Table S4** - Baseline characteristics and outcome events of patients with and without on-treatment acute exacerbation of idiopathic pulmonary fibrosis |
| Page 15-16 | **Supplementary Table S5 -** Baseline characteristics and outcome events of patients with and without on-treatment mortality |
| Page 17 | **Supplementary Table S6 -** Results from the receiver-operative characteristic analysis on cut-off values of continuous-variable candidate predictors for the three adverse outcomes |
| Page 18 | **Supplementary Table S7** - Cox proportional-hazards regression and subdistribution hazard regression analyses of candidate risk factors for nintedanib-related hepatic injury |
| Page 19 | **Supplementary Table S8 -** Ordinal logistic regression analysis of candidate risk factors for nintedanib-related severe and recurrent hepatic injury |
| Page 20 | **Supplementary Table S9** - Cox proportional-hazards regression and subdistribution hazard regression analyses of candidate risk factors for on-treatment acute exacerbation |
| Page 21 | **Supplementary Table S10** - Cox proportional-hazards regression analyses of candidate risk factors for on-treatment mortality |
| Page 22-23 | **Supplementary Table S11 -** Comparison in baseline characteristics and outcome events of patients with plasma KL-6 ≥ or < 2.5 ng/mL |
| Page 24-28 | **Appendix A:** Supplementary Protocol - The processing of blood specimen and the protocol of enzyme-linked immunosorbent assay (ELISA) for measuring plasma levels of Krebs von den Lungen-6 (KL-6) and surfactant protein A (SPA) |

**Supplementary Figure S1 - Sensitivity analyses on the multi-variable Cox proportional-hazards regression models**

**
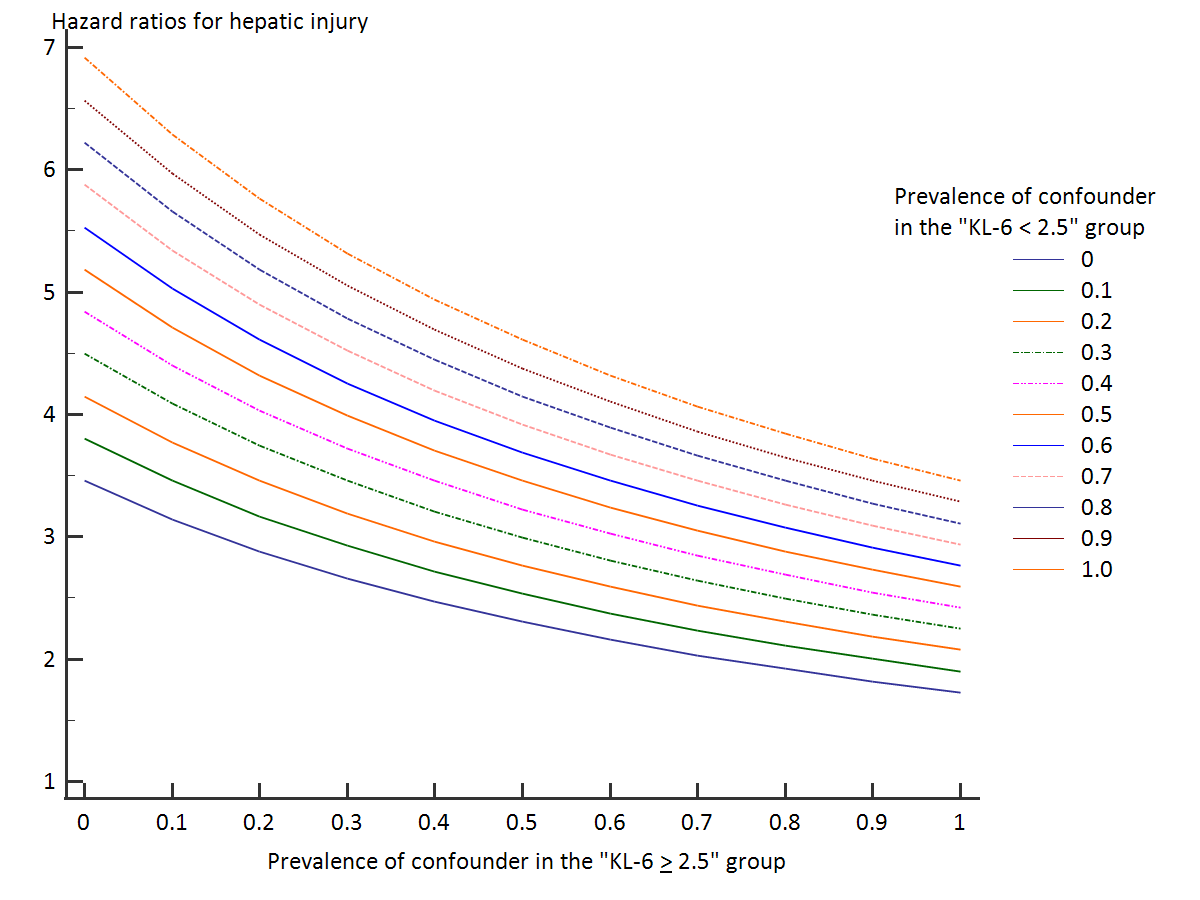
**

**Supplementary Figure S1a**

**
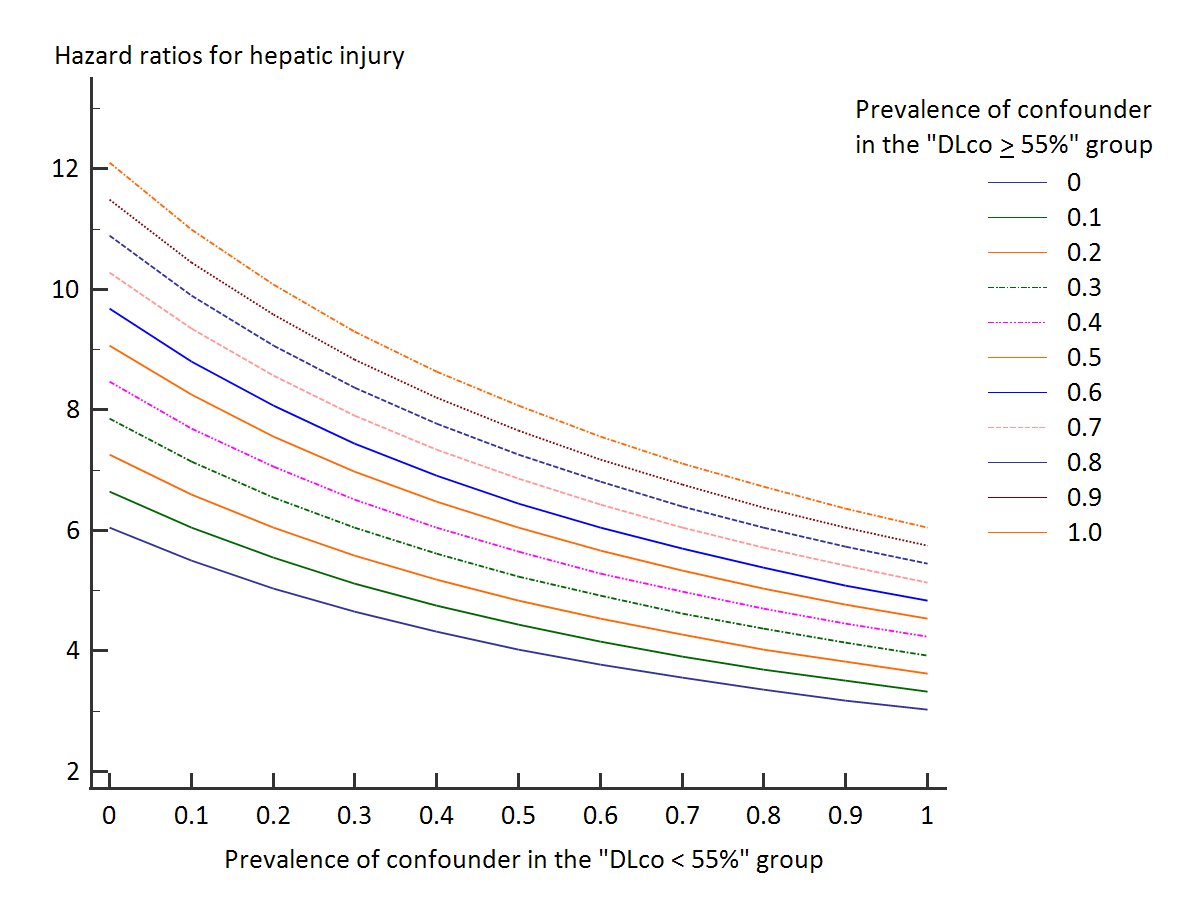
**

**Supplementary Figure S1b**


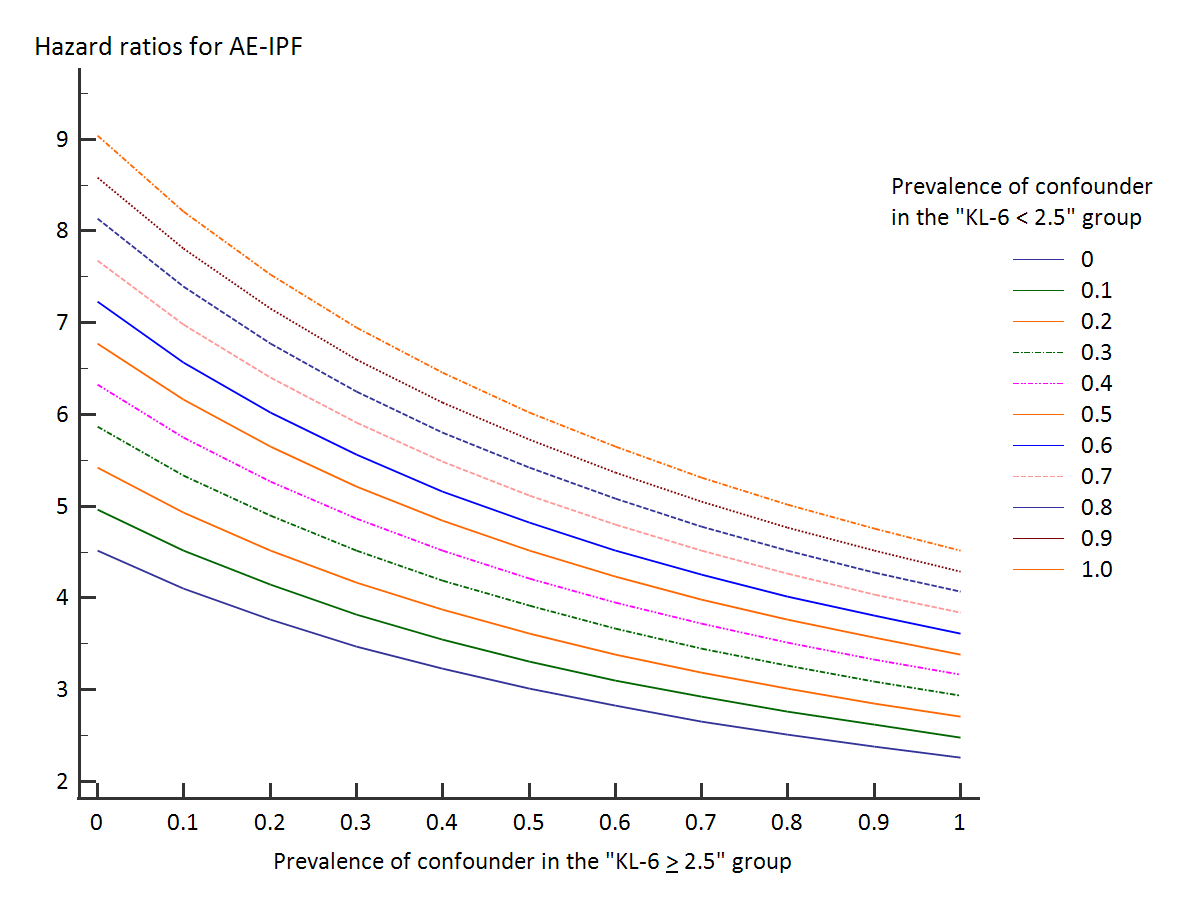


**Supplementary Figure S1c**


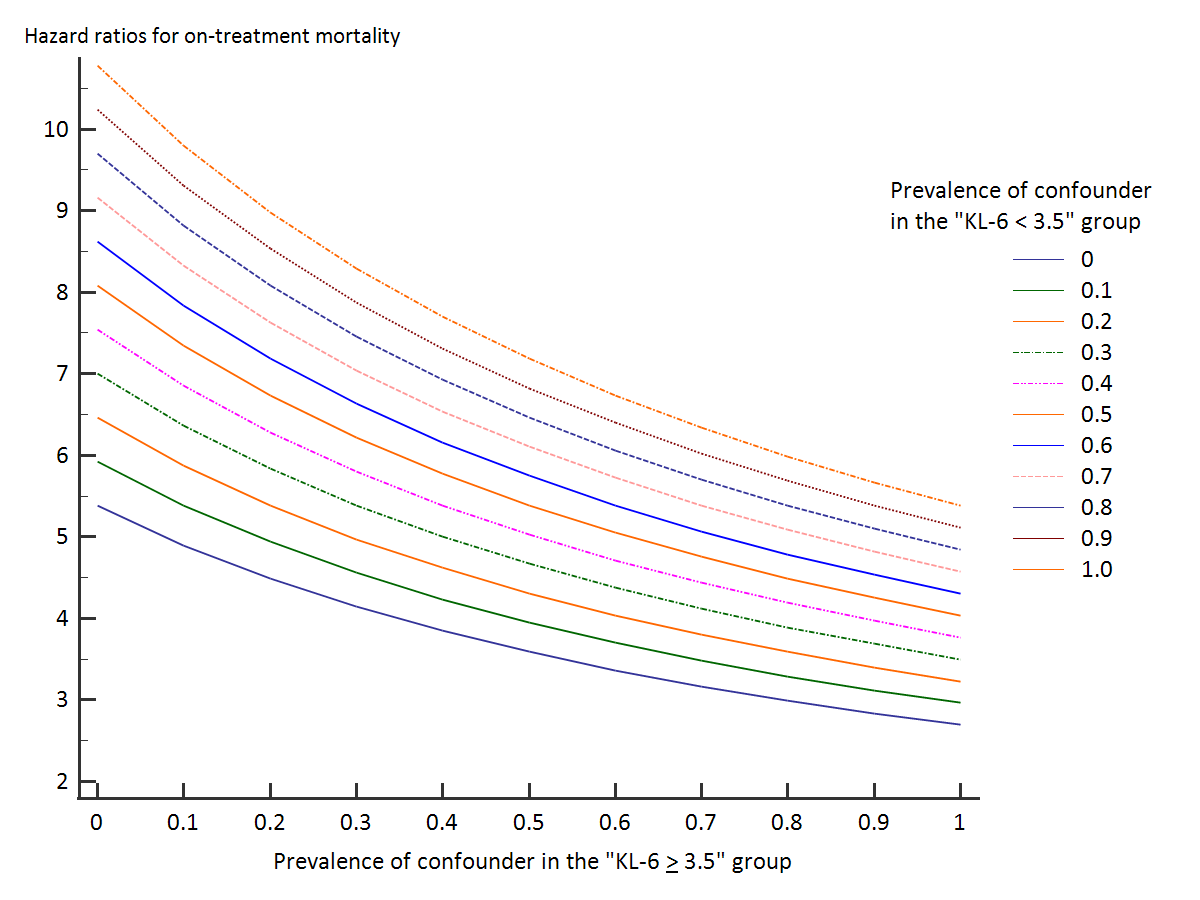


**Supplementary Figure S1d**


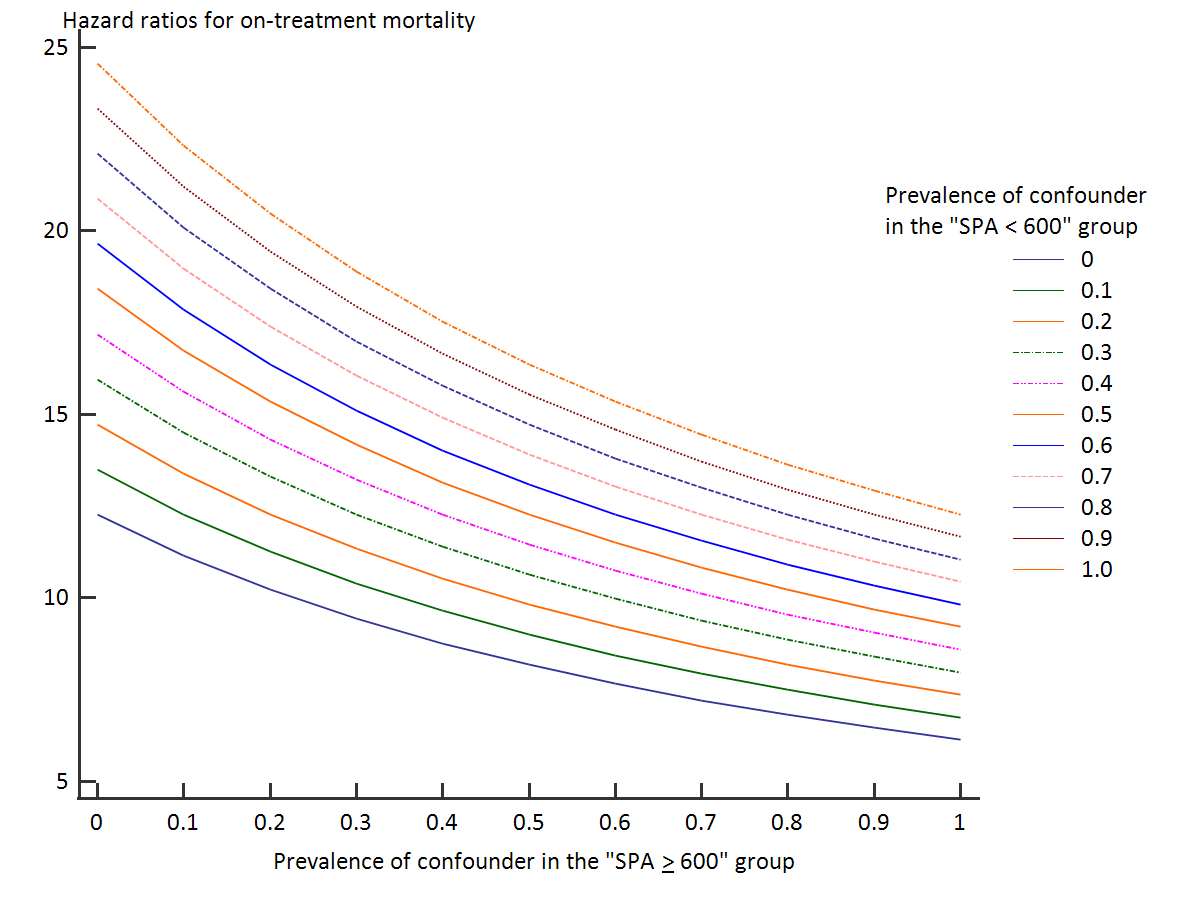


**Supplementary Figure S1e**

**Supplementary Figure S1 Explanation:**

The results of the sensitivity analyses were presented graphically in Figures S1a to S1e above, which show that, regardless of the various ratios of prevalence of the potentially unidentified confounder, baseline plasma KL-6 level ≥ 2.5 ng/mL (a) and baseline D_LCO_ < 55% predicted (b) persist as significant predictors for nintedanib-related hepatic injury; KL-6 ≥ 2.5 ng/mL remains a significant predictor for on-treatment AE-IPF (c); KL-6 ≥ 3.5 ng/mL (d) and SPA ≥ 600 pg/mL (e) persist as significant predictors for on-treatment mortality.

For example, as in Figure S1a, hypothetically when all the patients with baseline KL-6 < 2.5 ng/mL have the potentially unidentified confounder (and thus the prevalence of this confounder = 1.0, as represented by the top orange broken line), but none of the patients with baseline KL-6 ≥ 2.5 ng/mL have this unidentified confounder (and thus the prevalence of this confounder = 0), then KL-6 ≥ 2.5 ng/mL would still be a significant predictor for nintedanib-related hepatitis injury (with the adjusted hazard ratio = 6.915).

Sensitivity analyses were performed using R (Version 3.6.1) and the packages *survival* and *obsSens*; the graphs were plotted using MedCal (Version 16.8.4, MedCal Software, Belgium).

**Supplementary Figure S1 Abbreviation:** AE-IPF, acute exacerbation of idiopathic pulmonary fibrosis; D_LCO_, diffusion capacity for carbon monoxide; KL-6, Krebs von den Lungen-6; SPA, surfactant protein A.

**Supplementary Figure S2 – Comparison of pulmonary functional decline rates between patient groups stratified by the plasma Krebs von den Lungen-6 levels** ≥ **or < 3.5 ng/mL**


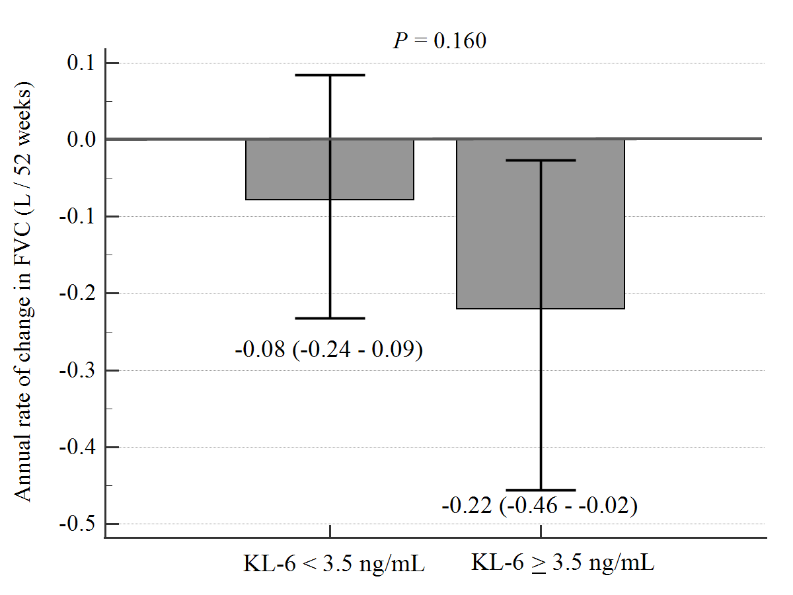

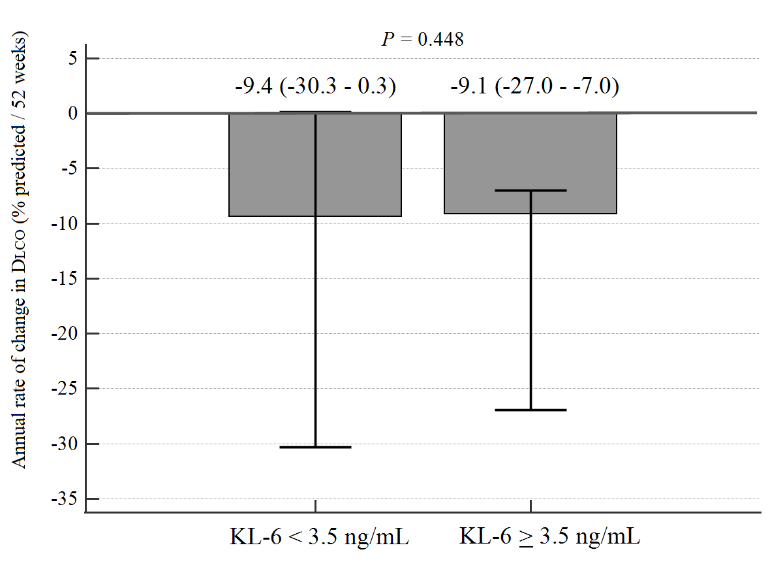


**Figure S2a Figure S2b**


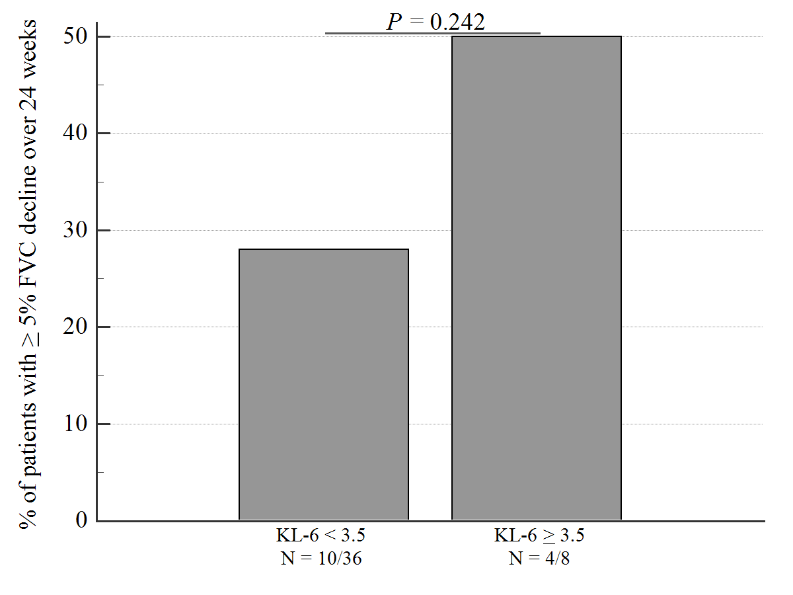

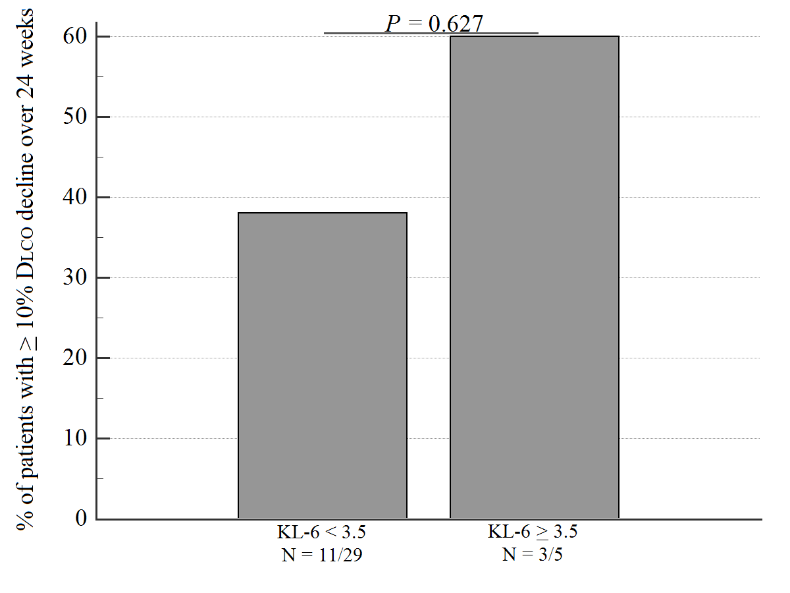


**Figure S2c Figure S2d**

**Supplementary Figure S2 Explanation:**

Comparison of patients having baseline plasma KL-6 < 3.5 ng/mL with patients having KL-6 ≥ 3.5 ng/mL in: (a) annual rate of FVC decline; (b) annual rate of DLco decline; (c) proportion of patients with ≥ 5% FVC decline over 24 weeks; (d) proportion of patients with ≥ 10% DLco decline over 24 weeks. (Note: The analysis was performed using Mann Whitney U test (panels a to b) and Fisher’s exact test (panels c to d). The analysis could **not** be performed after excluding patients with on-treatment acute exacerbation due to **too few** the number of cases remaining in each group.)

**Supplementary Figure S2 Abbreviation:** D_LCO_, diffusion capacity for carbon monoxide; FVC, forced vital capacity; KL-6, Krebs von den Lungen-6.

**Supplementary Table S1 - Timing of onset of on-treatment adverse outcomes**

| Time interval after nintedanib initiated when the adverse outcome occurred | Number of patients having the adverse outcomes (% of total N) | | |
| --- | --- | --- | --- |
|  | Hepatic injury  (total N = 24) | On-treatment AE-IPF  (total N = 20) | On-treatment mortality  (total N = 16) |
| Day 1-30 | 9 (38) | 0 (0) | 1 (6) |
| Day 31-60 | 2 (8) | 2 (10) | 1 (6) |
| Day 61-90 | 4 (17) | 1 (5) | 0 (0) |
| Day 91-180 | 4 (17) | 5 (25) | 1 (6) |
| After day 181 | 5 (20) | 12 (60) | 13 (82) |
| Day 1-60 | 11 (46) | 2 (10) | 2 (13) |
| Day 1-90 | 15 (63) | 3 (15) | 2 (13) |
| Day 1-180 | 19 (79) | 8 (40) | 3 (19) |
| Within 1 year | 24 (100) | 13 (65) | 6 (38) |
| Within 2 years | 24 (100) | 18 (90) | 11 (69) |
| Within 3 years | 24 (100) | 20 (100) | 14 (88) |
| Within 4 years | 24 (100) | 20 (100) | 16 (100) |
| AE-IPF, acute exacerbation of idiopathic pulmonary fibrosis. | | | |

**Supplementary Table S2 - Baseline characteristics and outcome events of patients with and without nintedanib-related hepatic injury**

| Baseline characteristics and outcome events | No hepatic injury  (n = 33) | Hepatic injury  (n = 24) | *P* – value^a^ |
| --- | --- | --- | --- |
| Age, years | 76.7 ± 9.9 | 73.6 ± 8.5 | 0.137 |
| Sex Female, n (%) | 5 (15) | 4 (17) | 1.000 |
| Male, n (%) | 28 (85) | 20 (83) |  |
| Body height, cm | 161.8 ± 8.0 | 162.8 ± 7.5 | 0.588 |
| Body weight, kg | 63.6 ± 9.6 | 63.3 ± 12.4 | 0.897 |
| Body mass index, kg/m^2^ | 24.4 ± 3.2 | 23.9 ± 4.9 | 0.279 |
| Patients with body mass index < 22 kg/m^2^, n (%) | 7 (21) | 8 (33) | 0.368 |
| Body surface area, m^2^ | 1.67 ± 0.15 | 1.67 ± 0.17 | 0.765 |
| Patients with body surface area < 1.58 m^2^, n (%) | 10 (30) | 8 (33) | 1.000 |
| Charlson comorbidity index | 5 (4-7) | 5 (3-6) | 0.372 |
| Chronic hepatitis B, n (%) | 4 (12) | 2 (8) | 1.000 |
| Chronic hepatitis C, n (%) | 4 (12) | 1 (4) | 0.385 |
| Other non-viral liver condition, n (%) | 1 (3) | 2 (8) | 0.567 |
| Fatty liver on baseline sonography, n (%) | 9 (27) | 6 (25) | 1.000 |
| Echocardiographic evidence of pulmonary  hypertension, n (%) | 17 (52) | 19 (79) | 0.051 |
| Echocardiographic evidence of LV dysfunction, n (%) | 0 (0) | 2 (8) | 0.173 |
| Dyslipidaemia, n (%) | 14 (42) | 12 (50) | 0.601 |
| Cigarette smoking status: |  |  | 0.489 |
| Never smoker, n (%) | 13 (39) | 13 (54) |  |
| Current smoker, n (%) | 3 (9) | 1 (4) |  |
| Former smoker, n (%) | 17 (52) | 10 (42) |  |
| Baseline plasma KL-6 level, ng/mL | 0.94 (0.44-1.63) | 2.72 (1.82-4.05) | < 0.001 |
| Baseline plasma SPA level, pg/mL | 238.5  (142.3-398.9) | 405.4  (197.9-484.6) | 0.068 |
| Baseline oximetry breathing ambient air, % | 95 (94-97) | 94 (92-97) | 0.078 |
| Baseline D_LCO_, mmol/min/kPa | 2.86 (2.29-4.25) | 2.58 (2.08-3.76) | 0.212 |
| Baseline D_LCO_, % predicted | 60 (53-83) | 42 (31-54) | 0.001 |
| Baseline FVC, L | 1.98 ± 0.38 | 2.06 ± 0.61 | 0.777 |
| Baseline FVC, % predicted | 67 ± 11 | 67 ± 14 | 0.808 |
| Stages based on the GAP index |  |  | 0.395 |
| Stage 1, n (%) | 8 (24) | 6 (25) |  |
| Stage 2, n (%) | 20 (61) | 11 (46) |  |
| Stage 3, n (%) | 5 (15) | 7 (29) |  |
| Dosing of nintedanib when hepatic injury |  |  | 1.000 |
| Full dose, n (%) | 25 (76) | 19 (79) |  |
| Reduced dose , n (%) | 8 (24) | 5 (21) |  |
| Severity of ALT elevation |  |  | - |
| No elevation, n (%) | 33 (100) | - |  |
| < 3 folds, n (%) | - | 16 (67) |  |
| > 3 and < 5 folds, n (%) | - | 5 (21) |  |
| > 5 and < 10 folds, n (%) | - | 2 (8) |  |
| > 10 folds, n (%) | - | 1 (4) |  |
| Duration of nintedanib treatment, days | 378 ± 410 | 484 ± 337 | 0.100 |
| Time to the first check of liver enzymes, days | 14 (14-20) | 14 (13-18) | 0.132 |
| Time to the first event of hepatic injury, days | - | 69 (17-156) | - |
| Time between plasma sampling and nintedanib initiation, days | 13 (0-25) | 5 (0-25) | 0.612 |
| Time between baseline pulmonary functions and nintedanib initiation, days | 28 (20-50) | 27 (14-83) | 0.716 |
| Time between plasma sampling and baseline pulmonary functions, days | 25 (14-86) | 18 (1-65) | 0.282 |
| On-treatment AE-IPF, n (%) | 5 (15) | 15 (63) | 0.001 |
| On-treatment mortality, n (%) | 6 (18) | 10 (42) | 0.074 |
| AE-IPF, acute exacerbation of idiopathic pulmonary fibrosis; ALT, alanine transaminase; D_LCO_, diffusion capacity for carbon monoxide; FVC, forced vital capacity; GAP, gender, age, physiology; KL-6, Krebs von den Lungen-6; LV, left ventricular; SPA, surfactant protein A. ^a^ *P*-value for the comparison of each variable between hepatic injury and no-hepatic injury groups. | | | |

**Supplementary Table S3 - Medications used concurrently to nintedanib treatment by patients with and without hepatic injury**

| Agents that were taken concurrently with nintedanib | Number of patients taking the agents  (% of N) | | *P*-value for the comparison between patients with and without hepatic injury ^a^ |
| --- | --- | --- | --- |
|  | No hepatic injury (N=33) | Hepatic injury  (N=24) |  |
| Acetaminophen | 6 (18) | 2 (8) | 0.446 |
| N-acetylcystein | 13 (39) | 8 (33) | 0.782 |
| Alprazolam | 2 (6) | 2 (8) | 1.000 |
| Ambroxol | 16 (48) | 9 (38) | 0.433 |
| Amiodarone | 0 (0) | 1 (4) | 0.421 |
| Amlodipine | 6 (18) | 1 (4) | 0.220 |
| Aspirin | 2 (6) | 3 (13) | 0.640 |
| Atorvastatin | 1 (3) | 1 (4) | 1.000 |
| Baclofen | 1 (3) | 1 (4) | 1.000 |
| Bisoprolol | 4 (12) | 1 (4) | 0.385 |
| Brotizolam | 0 (0) | 2 (8) | 0.173 |
| Bumetanide | 1 (3) | 0 (0) | 1.000 |
| Chlorzoxazone | 1 (3) | 1 (4) | 1.000 |
| Ciprofloxacin | 2 (6) | 1 (4) | 1.000 |
| Clopidogrel | 2 (6) | 3 (13) | 0.640 |
| Codeine | 2 (6) | 0 (0) | 0.504 |
| Dextromethorphan | 23 (70) | 15 (63) | 0.584 |
| Digoxin | 1 (3) | 0 (0) | 1.000 |
| Diltiazem | 2 (6) | 4 (17) | 0.227 |
| Dipyridamole | 1 (3) | 0 (0) | 1.000 |
| Dronedarone | 1 (3) | 0 (0) | 1.000 |
| Esomeprazole | 2 (6) | 0 (0) | 0.504 |
| Estazolam | 1 (3) | 0 (0) | 1.000 |
| Famotidine | 19 (56) | 13 (54) | 1.000 |
| Febuxostat | 2 (6) | 0 (0) | 0.504 |
| Fexofenadine | 7 (21) | 3 (13) | 0.494 |
| Fluvastatin | 1 (3) | 2 (8) | 0.567 |
| Furosemide | 4 (12) | 6 (25) | 0.294 |
| Glimepiride | 2 (6) | 2 (8) | 1.000 |
| Hydralazine | 0 (0) | 1 (4) | 0.421 |
| Hydroxyzine | 4 (12) | 0 (0) | 0.130 |
| Imipramine | 1 (3) | 0 (0) | 1.000 |
| Indapamide | 1 (3) | 0 (0) | 1.000 |
| Supplementary Table S3 continues | | | |
| Agents that were taken concurrently with nintedanib | No hepatic injury (N=33) | Hepatic injury  (N=24) | *P*-value for the comparison between patients with and without hepatic injury ^a^ |
| Irbesartan | 2 (6) | 0 (0) | 0.504 |
| Isosorbide mononitrate | 0 (0) | 2 (8) | 0.173 |
| Lercanidipine | 0 (0) | 1 (4) | 0.421 |
| Levofloxacin | 1 (3) | 0 (0) | 1.000 |
| Loperamide | 22 (67) | 10 (42) | 0.104 |
| Loratadine | 1 (3) | 1 (4) | 1.000 |
| Lorazepam | 2 (6) | 1 (4) | 1.000 |
| Medroxyprogesterone | 0 (0) | 2 (8) | 0.173 |
| Meloxicam | 0 (0) | 1 (4) | 0.421 |
| Metformin | 3 (9) | 1 (4) | 0.631 |
| Methylprednisolone | 2 (6) | 3 (13) | 0.640 |
| Metoclopramide | 2 (6) | 0 (0) | 0.504 |
| Mosapride | 9 (27) | 2 (8) | 0.097 |
| Montelukast | 1 (3) | 0 (0) | 1.000 |
| Nicorandil | 2 (6) | 0 (0) | 0.504 |
| Nifedipine | 3 (9) | 0 (0) | 0.256 |
| Pentoxifylline | 2 (6) | 0 (0) | 0.504 |
| Pioglitazone | 1 (3) | 0 (0) | 1.000 |
| Pitavastatin | 1 (3) | 0 (0) | 1.000 |
| Prednisolone | 10 (30) | 8 (33) | 1.000 |
| Procaterol | 0 (0) | 1 (4) | 0.421 |
| Ramipril | 0 (0) | 1 (4) | 0.421 |
| Repaglinide | 2 (6) | 2 (8) | 1.000 |
| Rivaroxaban | 3 (9) | 0 (0) | 0.256 |
| Rosuvastatin | 3 (9) | 1 (4) | 0.631 |
| Silodosin | 0 (0) | 1 (4) | 0.421 |
| Sitagliptin | 1 (3) | 3 (13) | 0.300 |
| Spironolactone | 2 (6) | 3 (13) | 0.640 |
| Solifenacin | 1 (3) | 0 (0) | 1.000 |
| Theophyllines | 9 (27) | 10 (42) | 0.273 |
| Trimethoprim/SMX | 2 (6) | 2 (8) | 1.000 |
| Tamsulosin | 4 (12) | 4 (17) | 0.709 |
| Terazosin | 1 (3) | 1 (4) | 1.000 |
| Thiamine | 0 (0) | 1 (4) | 0.421 |
| Tramadol | 1 (3) | 0 (0) | 1.000 |
| Supplementary Table S3 continues | | | |
| Agents that were taken concurrently with nintedanib | No hepatic injury (N=33) | Hepatic injury  (N=24) | *P*-value for the comparison between patients with and without hepatic injury ^a^ |
| Verapamil | 0 (0) | 2 (8) | 0.173 |
| Warfarin | 1 (3) | 0 (0) | 1.000 |
| Note: A medical agent was selected for analysis if it was taken by one of the included patients for at least 3 doses concurrently with nintedanib and within 2 weeks of each blood check for hepatic enzymes.  ^a^ For each medical agent, counts between the groups were analyzed using Fischer’s exact test. | | | |

**Supplementary Table S4 - Baseline characteristics and outcome events of patients with and without on-treatment acute exacerbation of idiopathic pulmonary fibrosis**

| Baseline characteristics and outcomes | No AE-IPF  (N = 37) | With AE-IPF  (N = 20) | *P* – value^a^ |
| --- | --- | --- | --- |
| Age, years | 76.4 ± 9.2 | 73.5 ± 9.7 | 0.300 |
| Sex Female, n (%) | 7 (19) | 2 (10) | 0.471 |
| Male n (%) | 30 (81) | 18 (90) |  |
| Body height, cm | 162.7 ± 8.1 | 161.3 ± 7.0 | 0.725 |
| Body weight, Kg | 63.7 ± 10.4 | 63.0 ± 11.7 | 0.676 |
| Body mass index, Kg/m^2^ | 24.2 ± 3.4 | 24.3 ± 4.9 | 0.593 |
| Body surface area, cm^2^ | 1.68 ± 0.16 | 1.66 ± 0.15 | 0.676 |
| Charlson comorbidity index | 5 (4-7) | 5 (3-6) | 0.360 |
| Echocardiographic evidence of LV dysfunction, n (%) | 1 (3) | 1 (5) | 1.000 |
| Echocardiographic evidence of pulmonary hypertension, n (%) | 20 (54) | 16 (80) | 0.084 |
| Dyslipidaemia, n (%) | 16 (43) | 10 (50) | 0.781 |
| Cigarette smoking status: |  |  | 0.141 |
| Never smoker, n (%) | 14 (38) | 12 (60) |  |
| Current smoker, n (%) | 4 (11) | 0 (0) |  |
| Former smoker, n (%) | 19 (51) | 8 (40) |  |
| Baseline plasma KL-6 level, ng/mL | 1.03 (0.48-1.86) | 3.11 (1.38-5.07) | 0.001 |
| Baseline plasma SPA level, pg/mL | 235.6  (157.3-379.9) | 412.6  (181.8-478.5) | 0.042 |
| Baseline pulse oximetry breathing ambient air, % | 95 (94-97) | 95 (93-97) | 0.254 |
| Baseline D_LCO_, mmol/min/kPa | 2.86 (2.19-4.06) | 2.51 (2.08-3.92) | 0.574 |
| Baseline D_LCO_, % predicted | 58 (42-73) | 50 (30-67) | 0.172 |
| Baseline FVC, L | 2.06 ± 0.50 | 1.94 ± 0.46 | 0.467 |
| Baseline FVC, % predicted | 69 ± 12 | 64 ± 12 | 0.195 |
| Stages based on the GAP index |  |  | 0.463 |
| Stage 1, n (%) | 10 (27) | 4 (20) |  |
| Stage 2, n (%) | 21 (57) | 10 (50) |  |
| Stage 3, n (%) | 6 (16) | 6 (30) |  |
| Dosing when on-treatment AE-IPF or censoring |  |  | 1.000 |
| Full dose, n (%) | 25 (68) | 14 (70) |  |
| Reduced dose, n (%) | 12 (32) | 6 (30) |  |
| Duration of nintedanib treatment, days | 345 ± 352 | 565 ± 401 | 0.022 |
| Time to first on-treatment AE-IPF, days | - | 238 (111-431) | - |
| Time between plasma sampling and nintedanib initiation, days | 2 (0-28) | 10 (0-28) | 0.951 |
| Time between baseline pulmonary functions and nintedanib initiation, days | 27 (15-50) | 30 (17-83) | 0.664 |
| Time between plasma sampling and baseline pulmonary functions, days | 25 (14-86) | 18 (2-65) | 0.558 |
| Nintedanib-related hepatic injury, n (%) | 9 (24) | 15 (75) | 0.001 |
| On-treatment mortality, n (%) | 4 (11) | 12 (60) | < 0.001 |
| AE-IPF, acute exacerbation of idiopathic pulmonary fibrosis; D_LCO_, diffusion capacity for carbon monoxide; FVC, forced vital capacity; GAP, gender, age, physiology; KL-6, Krebs von den Lungen-6; LV, left ventricular; SPA, surfactant protein A. ^a^ *P*-value for the comparison of each variable between patients with and without AE-IPF. | | | |

**Supplementary Table S5 - Baseline characteristics and outcome events of patients with and without on-treatment mortality**

| Baseline characteristics and outcomes | Survived  (N = 41) | Deceased  (N = 16) | *P* – value^a^ |
| --- | --- | --- | --- |
| Age, years | 75.1 ± 9.1 | 76.2 ± 10.4 | 0.534 |
| Sex Female, n (%) | 8 (20) | 1 (6) | 0.420 |
| Male, n (%) | 33 (80) | 15 (94) |  |
| Body height, cm | 162.6 ± 8.2 | 161.2 ± 6.5 | 0.575 |
| Body weight, Kg | 64.7 ± 11.1 | 60.3 ± 9.4 | 0.180 |
| Body mass index, Kg/m^2^ | 24.6 ± 4.1 | 23.2 ± 3.6 | 0.153 |
| Body surface area, cm^2^ | 1.69 ± 0.16 | 1.63 ± 0.13 | 0.201 |
| Charlson comorbidity index | 5 (4-7) | 6 (3-6) | 0.494 |
| Echocardiographic evidence of LV dysfunction, n (%) | 2 (5) | 0 (0) | 1.000 |
| Echocardiographic evidence of pulmonary hypertension, n (%) | 22 (54) | 14 (88) | 0.030 |
| Dyslipidaemia, n (%) | 20 (49) | 6 (38) | 0.558 |
| Cigarette smoking status: |  |  | 0.430 |
| Never smoker, n (%) | 18 (44) | 8 (50) |  |
| Current smoker, n (%) | 4 (10) | 0 (0) |  |
| Former smoker, n (%) | 19 (46) | 8 (50) |  |
| Baseline plasma KL-6 level, ng/mL | 1.31 (0.48-2.20) | 3.61 (1.28-8.22) | 0.001 |
| Baseline plasma SPA level, pg/mL | 226.0  (140.2-373.7) | 447.9  (393.5-697.3) | < 0.001 |
| Baseline pulse oximetry breathing ambient air, % | 95 (93-97) | 96 (93-97) | 0.823 |
| Baseline D_LCO_, mmol/min/kPa | 2.95 (2.21-3.95) | 2.41 (2.08-3.99) | 0.346 |
| Baseline D_LCO_, % predicted | 55 (39-70) | 55 (36-73) | 0.941 |
| Baseline FVC, L | 2.00 ± 0.53 | 2.05 ± 0.37 | 0.638 |
| Baseline FVC, % predicted | 67 ± 13 | 67 ± 11 | 0.729 |
| Stages based on the GAP index |  |  | 0.965 |
| Stage 1, n (%) | 10 (24) | 4 (25) |  |
| Stage 2, n (%) | 22 (54) | 9 (56) |  |
| Stage 3, n (%) | 9 (22) | 3 (19) |  |
| Dosing when on-treatment mortality or censoring |  |  | 0.341 |
| Full dose, n (%) | 30 (73) | 9 (56) |  |
| Reduced dose , n (%) | 11 (27) | 7 (44) |  |
| Duration of nintedanib treatment, days | 387 ± 388 | 512 ± 361 | 0.192 |
| Time to on-treatment mortality, days | - | 486 (217-811) | - |
| Time between plasma sampling and nintedanib initiation, days | 13 (0-28) | 5 (0-28) | 0.809 |
| Time between baseline pulmonary functions and nintedanib initiation, days | 27 (15-51) | 30 (17-82) | 0.804 |
| Time between plasma sampling and baseline pulmonary functions, days | 25 (12-86) | 17 (6-51) | 0.534 |
| Nintedanib-related hepatic injury, n (%) | 14 (34) | 10 (63) | 0.074 |
| On-treatment AE-IPF, n (%) | 8 (20) | 12 (75) | < 0.001 |
| AE-IPF, acute exacerbation of idiopathic pulmonary fibrosis; D_LCO_, diffusion capacity for carbon monoxide; FVC, forced vital capacity; GAP, gender, age, physiology; KL-6, Krebs von den Lungen-6; LV, left ventricular; SPA, surfactant protein A. ^a^ *P*-value for the comparison of each variable between patients with and without on-treatment mortality. | | | |

**Supplementary Table S6 - Results from the receiver-operative characteristic analysis on cut-off values of continuous-variable candidate predictors for the three adverse outcomes**

| **Adverse outcomes** | **Candidate predictors and**  **cut-off values** | **Area-under-curve**  **(95% CI)** | **Sensitivity** | **Specificity** | **Accuracy** | **Youden’s index** |
| --- | --- | --- | --- | --- | --- | --- |
| Hepatic injury | KL-6 ≥ or < 2.5 ng/mL | 0.815  (0.684-0.908) | 87 | 76 | 81 | 0.630 |
|  | D_LCO_ ≥ or < 55%  predicted | 0.763  (0.629-0.867) | 70 | 82 | 77 | 0.525 |
|  | SPO_2_ | No optimal cut-off value was identified (all the areas-under-curve < 0.7); this candidate predictor was included for analysis as a continuous variable. | | | | |
|  | SPA | No optimal cut-off value was identified (all the areas-under-curve < 0.7); this candidate predictor was included for analysis as a continuous variable. | | | | |
| AE-IPF | KL-6 ≥ or < 2.5 ng/mL | 0.783  (0.650-0.883) | 75 | 82 | 79 | 0.566 |
|  | SPA | No optimal cut-off value was identified (all the areas-under-curve < 0.7); this candidate predictor was included for analysis as a continuous variable. | | | | |
| Mortality | KL-6 ≥ or < 3.5 ng/mL | 0.867  (0.747-0.944) | 89 | 84 | 86 | 0.733 |
|  | SPA ≥ or < 600 pg/mL | 0.780  (0.648-0.880) | 80 | 76 | 77 | 0.560 |
| AE-IPF, acute exacerbation of idiopathic pulmonary fibrosis; 95% CI, 95% confidence interval; D_LCO_, diffusion capacity for carbon monoxide; KL-6, Krebs von den Lungen-6; SPA, surfactant protein A; SPO_2_, pulse oximetry (while breathing ambient air). | | | | | | |

**Supplementary Table S7 - Cox proportional-hazards regression and subdistribution hazard regression analyses of candidate risk factors for nintedanib-related hepatic injury**

| Candidate risk factors at baseline | Crude HR^a^ for  hepatic injury (95% CI) | *P*-value | Adjusted HR^a^ for hepatic injury  (95% CI) | *P*-value | Subdistribution  HR^b^ for hepatic injury (95% CI) | *P*-value |
| --- | --- | --- | --- | --- | --- | --- |
| Plasma KL-6 ≥ 2.5 ng/mL | 4.41 (1.87-10.40) | <0.001 | 3.46 (1.13-10.60) | 0.029 | 3.11 (1.00-9.74) | 0.051 |
| D_LCO_ < 55% predicted | 5.86 (2.15-15.93) | <0.001 | 6.05 (1.89-19.32) | 0.002 | 7.03 (2.37-20.80) | <0.001 |
| Baseline plasma SPA level ^c^ | 1.00 (0.99-1.00) | 0.256 |  |  |  |  |
| Baseline pulse oximetry while breathing ambient air ^c^ | 0.94 (0.86-1.03) | 0.197 |  |  |  |  |
| Echocardiographic evidence of pulmonary hypertension | 2.62 (0.98-7.02) | 0.056 | 1.21 (0.35-4.19) | 0.765 | 1.18 (0.38-3.64) | 0.770 |
| Body mass index < 22 kg/m^2 d^ | 1.84 (0.78-4.34) | 0.164 |  |  |  |  |
| Body surface area < 1.58 m^2 e^ | 1.34 (0.57-3.15) | 0.500 |  |  |  |  |
| D_LCO_, diffusion capacity for carbon monoxide; HR, hazard ratio; KL-6, Krebs von den Lungen-6; SPA, surfactant protein A; 95% CI, 95% confidence interval. ^a^ Derived from univariate and multi-variable Cox proportional-hazards regression analysis; for the multi-variable analysis, further adjustment was also made for gender-age-physiology (GAP) stages, Charlson comorbidity index, and the duration of nintedanib treatment. ^b^ Derived from subdistribution hazard regression analysis that adjusted for GAP stages, Charlson comorbidity index, the duration of nintedanib treatment, and further controlled for the competing risk of on-treatment mortality. ^c^ Baseline plasma SPA level and pulse oximetry were included for analysis as continuous variables due to the lack of optimal cutoff values for hepatic injury. ^d^ The cut-off value was proposed by Ikeda et al (reference 26). ^e^ The cut-off value was proposed by Ikeda et al (reference 27). | | | | | | |

**Supplementary Table S8 - Ordinal logistic regression analysis of candidate risk factors for nintedanib-related severe and recurrent hepatic injury**

| Candidate risk factors at baseline | Severe hepatic injury ^a^ | | | |
| --- | --- | --- | --- | --- |
|  | Crude OR^a^  (95% CI) | *P*-value | Adjusted OR^a^  (95% CI) | *P*-value |
| KL-6 ≥ 2.5 ng/mL | 9.94 (3.00-36.89) | <0.001 | 9.58 (1.97-55.67) | 0.007 |
| D_LCO_ < 55% predicted | 10.73 (3.30-40.86) | <0.001 | 10.89 (2.63-54.25) | 0.002 |
| Baseline plasma SPA level ^b^ | 1.00 (0.99-1.00) | 0.186 |  |  |
| Baseline pulse oximetry breathing ambient air ^b^ | 0.90 (0.76-1.05) | 0.196 |  |  |
| Echocardiographic evidence  of pulmonary hypertension | 3.28 (1.06-11.64) | 0.049 | 0.90 (0.17-4.62) | 0.897 |
| BMI < 22 kg/m^2 c^ | 1.59 (0.51-4.84) | 0.418 |  |  |
| BSA < 1.58 m^2 d^ | 1.19 (0.39-3.53) | 0.753 |  |  |
| Candidate risk factors at baseline | Recurrent hepatic injury ^e^ | | | |
|  | Crude OR^e^  (95% CI) | *P*-value | Adjusted OR^e^  (95% CI) | *P*-value |
| KL-6 ≥ 2.5 ng/mL | 10.21 (3.04-38.13) | <0.001 | 26.01 (4.19-260.13) | 0.001 |
| D_LCO_ < 55% predicted | 9.45 (2.95-35.17) | <0.001 | 60.28 (8.30-823.62) | <0.001 |
| Baseline plasma SPA level ^b^ | 1.00 (0.99-1.00) | 0.109 |  |  |
| Baseline pulse oximetry breathing ambient air ^b^ | 0.92 (0.77-1.07) | 0.295 |  |  |
| Echocardiographic evidence  of pulmonary hypertension | 3.07 (0.99-10.86) | 0.062 | 1.22 (0.23-6.81) | 0.813 |
| BMI < 22 kg/m^2 c^ | 1.83 (0.55-5.94) | 0.313 |  |  |
| BSA < 1.58 m^2 d^ | 1.01 (0.32-3.05) | 0.984 |  |  |
| BMI, body mass index; BSA, body surface area; D_LCO_, diffusion capacity for carbon monoxide; KL-6, Krebs von den Lungen-6; OR, odds ratio; SPA, surfactant protein A; 95% CI, 95% confidence interval. ^a^ Analysis was done using univariate and multivariable ordinal logistic regression analysis wherein the outcome was divided into 3 levels: 1, no hepatic injury; 2, non-severe hepatic injury; 3, severe hepatic injury; for multi-variable analysis, further adjustment was also made for gender-age-physiology (GAP) stages, Charlson comorbidity index, and the duration of nintedanib treatment. ^b^ Baseline plasma SPA level and pulse oximetry were included for analysis as continuous variables due to the lack of optimal cut-off values for hepatic injury. ^c^ The cut-off value was proposed by Ikeda et al (reference 26). ^d^ The cut-off value was proposed by Ikeda et al (reference 27). ^e^ Analysis was done using univariate and multivariable ordinal logistic regression analysis wherein the outcome was divided into 3 levels: 1, no hepatic injury; 2, hepatic injury without recurrence; 3, hepatic injury with recurrence; for multi-variable analysis, further adjustment was also made for GAP stages, Charlson comorbidity index, and the duration of nintedanib treatment. | | | | |

**Supplementary Table S9 - Cox proportional-hazards regression and subdistribution hazard regression analyses of candidate risk factors for on-treatment acute exacerbation**

| Candidate risk factors at baseline | Crude HR^a^ for  AE-IPF (95%CI) | *P*-value | Adjusted HR^a^ for AE-IPF  (95%CI) | *P*-value | Subdistribution  HR^b^ for AE-IPF  (95%CI) | *P*-value |
| --- | --- | --- | --- | --- | --- | --- |
| Plasma KL-6 ≥ 2.5 ng/mL | 4.15 (1.62-10.63) | 0.003 | 4.52 (1.63-12.55) | 0.004 | 3.54 (1.47-8.54) | 0.005 |
| Baseline plasma SPA level ^c^ | 1.00 (0.99-1.00) | 0.111 |  |  |  |  |
| Echocardiographic evidence of pulmonary hypertension | 2.23 (0.74-6.68) | 0.153 |  |  |  |  |
| AE-IPF, (on-treatment) acute exacerbation of idiopathic pulmonary fibrosis; HR, hazard ratio; KL-6, Krebs von den Lungen-6; 95%CI, 95% confidence interval; SPA, surfactant protein A. ^a^ Derived from univariate and multi-variable Cox proportional-hazards regression analysis; for the multi-variable analysis, further adjustment was also made for gender-age-physiology (GAP) stages, Charlson comorbidity index, and the duration of nintedanib treatment. ^b^ Derived from subdistribution hazard regression analysis that adjusted for GAP stages, Charlson comorbidity index, the duration of nintedanib treatment, and further controlled for the competing risk of on-treatment mortality. ^c^ Baseline plasma SPA level was included for analysis as a continuous variable due to the lack of optimal cut-off values for AE-IPF. | | | | | | |

| Candidate risk factors at baseline | Crude HR^a^ for  on-treatment mortality (95%CI) | *P*-value | Adjusted HR^a^ for  on-treatment mortality (95%CI) | *P*-value |
| --- | --- | --- | --- | --- |
| Plasma KL-6 ≥ 3.5 ng/mL | 4.23 (1.53-11.71) | 0.005 | 5.39 (1.16-24.97) | 0.031 |
| Plasma SPA ≥ 600 pg/mL | 14.17 (3.48-57.65) | <0.001 | 12.28 (2.06-73.05) | 0.006 |
| Echocardiographic evidence of pulmonary hypertension | 2.70 (0.60-12.13) | 0.194 |  |  |
| Having on-treatment AE-IPF | 3.23 (1.03-10.13) | 0.045 | 0.78 (0.16-3.88) | 0.765 |
| AE-IPF, acute exacerbation of idiopathic pulmonary fibrosis; HR, hazard ratio; KL-6, Krebs von den Lungen-6; SPA, surfactant protein A; 95%CI, 95% confidence interval. ^a^ Derived from univariate and multi-variable Cox proportional-hazards regression analysis; for the multi-variable analysis, further adjustment was also made for gender-age-physiology (GAP) stages, Charlson comorbidity index, and the duration of nintedanib treatment. | | | | |

**Supplementary Table S10 - Cox proportional-hazards regression analyses of candidate risk factors for on-treatment mortality**

**Supplementary Table S11 – Comparison in baseline characteristics and outcome events of patients with plasma KL-6 ≥ or < 2.5 ng/mL**

| Baseline characteristics and outcome events | KL-6 < 2.5 ng/mL  (n = 38)^a^ | KL-6 ≥ 2.5 ng/mL  (n = 16)^a^ | *P* – value^b^ |
| --- | --- | --- | --- |
| Age, years | 75.2 ± 9.6 | 75.0 ± 9.5 | 0.925 |
| Sex Female, n (%) | 7 (18) | 2 (13) | 0.709 |
| Male, n (%) | 31 (82) | 14 (87) |  |
| Body height, cm | 162.6 ± 8.1 | 160.9 ± 7.2 | 0.676 |
| Body weight, kg | 65.3 ± 10.1 | 58.1 ± 10.3 | 0.022 |
| Body mass index, kg/m^2^ | 24.9 ± 3.8 | 22.4 ± 3.6 | 0.054 |
| Body surface area, m^2^ | 1.70 ± 0.15 | 1.60 ± 0.15 | 0.034 |
| Charlson comorbidity index | 5 (3-7) | 5 (3-6) | 0.571 |
| Chronic hepatitis B, n (%) | 5 (13) | 1 (6) | 0.657 |
| Chronic hepatitis C, n (%) | 4 (11) | 1 (6) | 1.000 |
| Other non-viral liver condition, n (%) | 2 (5) | 1 (6) | 1.000 |
| Echocardiographic evidence of pulmonary  hypertension, n (%) | 18 (47) | 15 (94) | 0.002 |
| Echocardiographic evidence of LV dysfunction, n (%) | 0 (0) | 2 (13) | 0.084 |
| Cigarette smoking status: |  |  | 0.079 |
| Never smoker, n (%) | 14 (37) | 11 (69) |  |
| Current smoker, n (%) | 3 (8) | 0 (0) |  |
| Former smoker, n (%) | 21 (55) | 5 (31) |  |
| Baseline oximetry breathing ambient air, % | 96 (94-97) | 94 (90-97) | 0.159 |
| Baseline D_LCO_, mmol/min/kPa | 3.13 (2.26-4.21) | 2.13 (1.98-2.49) | 0.009 |
| Baseline D_LCO_, % predicted | 59 (47-74) | 37 (30-59) | 0.009 |
| Baseline FVC, L | 2.03 ± 0.49 | 2.02 ± 0.54 | 0.940 |
| Baseline FVC, % predicted | 68 ± 12 | 66 ± 14 | 0.762 |
| Stages based on the GAP index |  |  | 0.066 |
| Stage 1, n (%) | 11 (29) | 3 (19) |  |
| Stage 2, n (%) | 23 (60) | 7 (44) |  |
| Stage 3, n (%) | 4 (11) | 6 (37) |  |
| Dosing of nintedanib when hepatic injury |  |  | 1.000 |
| Full dose, n (%) | 29 (76) | 12 (75) |  |
| Reduced dose , n (%) | 9 (24) | 4 (25) |  |
| Dosing of nintedanib when AE-IPF |  |  | 0.351 |
| Full dose, n (%) | 27 (71) | 9 (56) |  |
| Reduced dose , n (%) | 11 (29) | 7 (44) |  |
| Dosing of nintedanib when mortality |  |  | 0.351 |
| Full dose, n (%) | 27 (71) | 9 (56) |  |
| Reduced dose , n (%) | 11 (29) | 7 (44) |  |
| Duration of nintedanib treatment | 399 ± 409 | 498 ± 320 | 0.185 |
| Time to first check of hepatic enzymes, days | 14 (14-15) | 16 (14-21) | 0.288 |
| Time between plasma sampling and nintedanib initiation, days | 7 (0-30) | 3 (0-25) | 0.734 |
| Time between baseline pulmonary functions and nintedanib initiation, days | 28 (15-43) | 28 (18-83) | 0.513 |
| Time between plasma sampling and baseline pulmonary functions, days | 21 (13-61) | 27 (6-95) | 0.583 |
| Nintedanib related hepatic injury, n (%) | 9 (24) | 13 (81) | <0.001 |
| On-treatment AE-IPF, n (%) | 7 (18) | 12 (75) | <0.001 |
| On-treatment mortality, n (%) | 6 (16) | 9 (56) | 0.006 |
| AE-IPF, acute exacerbation of idiopathic pulmonary fibrosis; ALT, alanine transaminase; D_LCO_, diffusion capacity for carbon monoxide; FVC, forced vital capacity; GAP, gender, age, physiology; KL-6, Krebs von den Lungen-6; LV, left ventricular; SPA, surfactant protein A. ^a^ Baseline plasma KL-6 level was not available from 3 of the 57 patients. ^b^ *P*-value for the comparison of each variable between hepatic injury and no-hepatic injury groups. | | | |

**Appendix A: Supplementary Protocol - The processing of blood specimen and the protocol of enzyme-linked immunosorbent assay (ELISA) for measuring plasma levels of Krebs von den Lungen-6 (KL-6) and surfactant protein A (SPA)**

**Part 1. The processing of blood specimen**

1. This study (including the experiments using human blood specimens) has been approved by the Institutional Review Board of the National Cheng Kung University Hospital (B-ER-105-390 and A-ER-107-193).
2. After informed consent was obtained, blood specimen (10 ml) was collected from the patient by specialized technicians from the Division of Clinical Pathology, Department of Pathology of National Cheng Kung University Hospital, using a EDTA vacuum collection tube.
3. The specimen was immediately ice-bathed, and quickly transferred to a specialized refrigerator, storing at 4°C.
4. Within 4 hours, the specimen was centrifuged (at 4 °C and 1000 xG, using Eppendorf Centrifuge 5810R) for 20 minutes.
5. After centrifugation, in a laminar flow bench and with aseptic maneuvers, the supernatant was aspirated, divided into sterile eppendorf tubes, and immediately stored at -80°C until use.

**Part 2. The protocol of enzyme-linked immunosorbent assay (ELISA) for measuring plasma levels of Krebs von den Lungen-6 (KL-6)**

1. We used a specialized kit from Fine Test (product umber: **EH0406**; manufactured by Wuhan Fine Biotech Co., Ltd, China) for conducting the ELISA.
2. The preparatory phase:
3. The kit was stored in a specialized refrigerator at 4°C, and was taken out and placed at room temperature 20 minutes before proceeding with subsequent steps.
4. The test sample: supernatant of the centrifuged plasma (as described in Part 1), stored at -80 °C, was gently thawed at 4 °C, and then diluted 2 folds (using the sample dilution buffer) immediately before the ELISA.
5. The wash buffer was well mixed, and then diluted 25 folds using sterile distilled water.
6. Lyophilized standard vials (each containing 10 ng of KL-6 standard; two vials were provided in each kit) was dissolved by adding 1 mL of the sample dilution buffer and then well mixed, yielding a concentration of 10 ng/mL. Sequential dilution was then performed (by mixing 300 uL of the higher concentration with 300 uL of sample dilution buffer), giving rise to a spectrum of standard solutions with progressively decreasing concentrations (namely, 10 🡪 5 🡪 2.5 🡪 1.25 🡪 0.625 🡪 0.313 🡪 0.156 🡪 0 ng/mL). Both vials of standard were used in each ELISA for duplication.
7. The 96-well microplate (already pre-coated with KL-6-specific primary antibodies) was unpacked and gently washed (350 uL per well) for 2 times (each time 1 minute 30 seconds) using the diluted wash buffer (prepared in (3)).
8. The reaction and reading phase:
9. 100 uL each of the standard solutions (with progressively decreasing concentrations as described above), test samples (triple repeats for each specimen), and controls (distilled water, sample dilution buffer) was added into individual specified wells of the micro-plate; the positions were carefully specified and recorded.
10. The loaded micro-plate was then covered and incubated at 37°C for 90 minutes.
11. After 90 minutes of incubation, the cover was removed and the well content carefully discarded. The micro-plate was washed 2 times (using the diluted wash buffer 350 uL / well and 1 min. 30 sec. / time).
12. 100 uL of diluted (1:100) biotin-labeled antibody (the secondary antibody; diluted 100 folds using the antibody dilution buffer as provided by the kit) was added into each well.
13. The micro-plate was then covered and incubated at 37°C for 60 minutes.
14. After 60 minutes of incubation, the cover was removed and the well content carefully discarded. The micro-plate was washed 3 times (using the diluted wash buffer 350 uL / well and 1 min. 30 sec. / time).
15. 100 uL of diluted (1:100) HRP-Streptavidin Conjugate (SABC) working solution (the tertiary antibody; diluted 100 folds using the SABC dilution buffer as provided by the kit) was added into each well.
16. The micro-plate was then covered and incubated at 37°C for 30 minutes.
17. After 30 minutes of incubation, the cover was removed and the well content carefully discarded. The micro-plate was washed 5 times (using the diluted wash buffer 350 uL / well and 1 min. 30 sec. / time).
18. In a dim condition, we added 90 uL of undiluted TMB (3,3’,5,5’-Tetramethylbenzidine) substrate into each well, and immediately covered the micro-plate and incubated at 37°C for 15-30 minutes. During this incubation, the cover was briefly and carefully removed (still in a dim condition) periodically to observe the change in coloration of the wells.
19. At the end of the incubation, we added 50 uL of undiluted Stop Solution into all the wells to terminate the TMB-reaction simultaneously.
20. The optical density absorbance at 450 nm of all the wells was immediately read, and the corresponding standard curve and concentrations of KL-6 were calculated, by a specialized micro-plate reader (SpectraMax 340PC384, run by Soft Max Pro Software, version 5.4.1) at the Core Laboratory of the Department of Clinical Medical Research of National Cheng Kung University Hospital, College of Medicine, National Cheng Kung University.
21. For quality control, the experiment for each specimen was repeated three times. An intra-assay CV (coefficient of variation) of < 15% and an inter-assay CV of < 15% was considered acceptable.

**Part 3. The protocol of enzyme-linked immunosorbent assay (ELISA) for measuring plasma levels of surfactant protein A (SPA)**

1. We used a specialized kit from Fine Test (product umber: **EH3806**; manufactured by Wuhan Fine Biotech Co., Ltd, China) for conducting the ELISA.
2. The preparatory phase:
   1. The kit was stored in a specialized refrigerator at 4°C, and was taken out and placed at room temperature 20 minutes before proceeding with subsequent steps.
   2. The test sample: supernatant of the centrifuged plasma (as described in Part 1), stored at -80 °C, was gently thawed at 4 °C, and then diluted 2 folds (using the sample dilution buffer) immediately before the ELISA.
   3. The wash buffer was well mixed, and then diluted 25 folds using sterile distilled water.
   4. Lyophilized standard vials (each containing 1000 pg of SPA standard; two vials were provided in each kit) was dissolved by adding 1 mL of the sample dilution buffer and then well mixed, yielding a concentration of 1000 pg/mL. Sequential dilution was then performed (by mixing 300 uL of the higher concentration with 300 uL of sample dilution buffer), giving rise to a spectrum of standard solutions with progressively decreasing concentrations (namely, 1000 🡪 500 🡪 250 🡪 125 🡪 62.5 🡪 31.3 🡪 15.6 🡪 0 pg/mL). Both vials of standard were used in each ELISA for duplication.
   5. The 96-well microplate (already pre-coated with SPA-specific primary antibodies) was unpacked and gently washed (350 uL per well) for 2 times (each time 1 minute 30 seconds) using the diluted wash buffer (prepared in (3)).
3. The reaction and reading phase:
4. 100 uL each of the standard solutions (with progressively decreasing concentrations as described above), test samples (triple repeats for each specimen), and controls (distilled water, sample dilution buffer) was added into individual specified wells of the micro-plate; the positions were carefully specified and recorded.
5. The loaded micro-plate was then covered and incubated at 37°C for 90 minutes.
6. After 90 minutes of incubation, the cover was removed and the well content carefully discarded. The micro-plate was washed 2 times (using the diluted wash buffer 350 uL / well and 1 min. 30 sec. / time).
7. 100 uL of diluted (1:100) biotin-labeled antibody (the secondary antibody; diluted 100 folds using the antibody dilution buffer as provided by the kit) was added into each well.
8. The micro-plate was then covered and incubated at 37°C for 60 minutes.
9. After 60 minutes of incubation, the cover was removed and the well content carefully discarded. The micro-plate was washed 3 times (using the diluted wash buffer 350 uL / well and 1 min. 30 sec. / time).
10. 100 uL of diluted (1:100) HRP-Streptavidin Conjugate (SABC) working solution (the tertiary antibody; diluted 100 folds using the SABC dilution buffer as provided by the kit) was added into each well.
11. The micro-plate was then covered and incubated at 37°C for 30 minutes.
12. After 30 minutes of incubation, the cover was removed and the well content carefully discarded. The micro-plate was washed 5 times (using the diluted wash buffer 350 uL / well and 1 min. 30 sec. / time).
13. In a dim condition, we added 90 uL of undiluted TMB (3,3’,5,5’-Tetramethylbenzidine) substrate into each well, and immediately cover the micro-plate and incubated at 37°C for 15-30 minutes. During this incubation, the cover would be briefly and carefully removed (still in a dim condition) periodically to observe the change in coloration of the wells.
14. At the end of the incubation, we added 50 uL of undiluted Stop Solution into all the wells to terminate the TMB-reaction simultaneously.
15. The optical density absorbance at 450 nm of all the wells was immediately read, and the corresponding standard curve and concentrations of SPA were calculated, by a specialized micro-plate reader (SpectraMax 340PC384, run by Soft Max Pro Software, version 5.4.1) at the Core Laboratory of the Department of Clinical Medical Research of National Cheng Kung University Hospital, College of Medicine, National Cheng Kung University.
16. For quality control, the experiment for each specimen was repeated three times. An intra-assay CV (coefficient of variation) of < 15% and an inter-assay CV of < 15% was considered acceptable.
